# Improving Malaria diagnosis through interpretable customized CNNs architectures

**DOI:** 10.1038/s41598-025-90851-1

**Published:** 2025-02-22

**Authors:** Md. Faysal Ahamed, Md Nahiduzzaman, Golam Mahmud, Fariya Bintay Shafi, Mohamed Arselene Ayari, Amith Khandakar, M. Abdullah-Al-Wadud, S. M. Riazul Islam

**Affiliations:** 1 Department of Electrical & Computer Engineering, Rajshahi University of Engineering & Technology, Rajshahi, 6204 Bangladesh; 2 Department of Civil & Environmental Engineering, Qatar University, 2713 Doha, Qatar; 3 Department of Electrical Engineering, Qatar University, 2713 Doha, Qatar; 4 Department of Software Engineering, College of Computer and Information Sciences, King Saud University, 11543 Riyadh, Saudi Arabia; 5 School of Natural and Computing Sciences, University of Aberdeen, Aberdeen, AB24 3FX UK

**Keywords:** Parallel convolutional neural network (PCNN), Soft Attention Parallel Convolutional Neural Networks (SPCNN), Soft Attention after Functional Block Parallel Convolutional Neural Networks (SFPCNN), Soft attention mechanism, Blood smear, Plasmodium parasite, Computational platforms and environments, Machine learning

## Abstract

Malaria, which is spread via female Anopheles mosquitoes and is brought on by the Plasmodium parasite, persists as a serious illness, especially in areas with a high mosquito density. Traditional detection techniques, like examining blood samples with a microscope, tend to be labor-intensive, unreliable and necessitate specialized individuals. To address these challenges, we employed several customized convolutional neural networks (CNNs), including Parallel convolutional neural network (PCNN), Soft Attention Parallel Convolutional Neural Networks (SPCNN), and Soft Attention after Functional Block Parallel Convolutional Neural Networks (SFPCNN), to improve the effectiveness of malaria diagnosis. Among these, the SPCNN emerged as the most successful model, outperforming all other models in evaluation metrics. The SPCNN achieved a precision of 99.38 $\pm$ 0.21%, recall of 99.37 $\pm$ 0.21%, F1 score of 99.37 $\pm$ 0.21%, accuracy of 99.37 ± 0.30%, and an area under the receiver operating characteristic curve (AUC) of 99.95 ± 0.01%, demonstrating its robustness in detecting malaria parasites. Furthermore, we employed various transfer learning (TL) algorithms, including VGG16, ResNet152, MobileNetV3Small, EfficientNetB6, EfficientNetB7, DenseNet201, Vision Transformer (ViT), Data-efficient Image Transformer (DeiT), ImageIntern, and Swin Transformer (versions v1 and v2). The proposed SPCNN model surpassed all these TL methods in every evaluation measure. The SPCNN model, with 2.207 million parameters and a size of 26 MB, is more complex than PCNN but simpler than SFPCNN. Despite this, SPCNN exhibited the fastest testing times (0.00252 s), making it more computationally efficient than both PCNN and SFPCNN. We assessed model interpretability using feature activation maps, Gradient-weighted Class Activation Mapping (Grad-CAM) and SHapley Additive exPlanations (SHAP) visualizations for all three architectures, illustrating why SPCNN outperformed the others. The findings from our experiments show a significant improvement in malaria parasite diagnosis. The proposed approach outperforms traditional manual microscopy in terms of both accuracy and speed. This study highlights the importance of utilizing cutting-edge technologies to develop robust and effective diagnostic tools for malaria prevention.

## Introduction

The Plasmodium parasite causes malaria and is one of the main contributors to morbidity and mortality in endemic parts of sub-Saharan Africa^1^. It mainly spreads to humans when a female Anopheles mosquito, already infected after feeding on a human, bites them. Particularly in areas where it is endemic—mostly in tropical regions where mosquitoes proliferate—the sickness can be fatal^2^. In accordance with the latest WHO World Malaria Report of 2023, approximately 249 million malaria cases are projected to have occurred in 85 malaria-endemic regions, including Nigeria, the Congo, Uganda, and various other tropical and humid areas. Approximately 249 million malaria cases are projected to have occurred in 85 malaria-endemic regions, including Nigeria, the Congo, Uganda, and various other tropical and humid areas, as reported in the latest WHO World Malaria Report of 2023^3^. Due to the rapid climate change in recent years, temperature, humidity, and rainfall fluctuate randomly, which can influence the behavior and survival of malaria-carrying Anopheles mosquitoes. In the sub-Saharan region of Africa, four types of malaria parasites typically coexist in overlapping geographic areas: Plasmodium ovale, P. falciparum, P. vivax, and P. malariae^4^. It is typical to see these parasites infecting identical populations simultaneously. Because of this, a single host may contract multiple infections, making it extremely difficult to quickly identify the malaria parasite and distinguish between multiple infections^5^. Plasmodium falciparum is the most common of these four species, accounting for most occurrences of severe malaria and fatalities. Malaria’s natural cycle involves transmission between human and female Anopheles mosquitos. Anopheles mosquitoes go through four phases in their life cycle: the egg, larva, pupa, and adult. Initially, the parasite develops and multiplies in the human liver before invading the red blood cells, causing symptoms. When mosquitoes feed on infected human blood, they ingest male and female parasites that reproduce further within the mosquito^6,7^. After development, the parasite migrates to the mosquito’s slivery glands in the form of sporozoites and transmits to another human through its bite. In most cases, typically, malaria takes 10 to 15 days to show symptoms following a bite by an infected mosquito. These symptoms are like flu symptoms: fever, headache, and chills, but they might be mild and may not be recognized as an indication of malaria. Individuals in tropical areas where malaria is common can become infected but remain asymptomatic because of partial immunity^8,9^.

Thin or thick blood smears are commonly employed in laboratory procedures such as microscopy, PCR, and RDT within the traditional malaria diagnosis framework^10^. The limitation of traditional techniques lies in their heavy dependence on the manual inspection of blood samples utilizing a microscope. Such methods are inherently subjective, require considerable time investment, and demand a high level of expertise from personnel. The accuracy and consistency of diagnosis are questioned due to the reliance on human clinical specialists. To overcome these limitations, there is a need for the development of automated, reliable, computer-based malaria diagnosis systems^2,11^.

The good news is that malaria isn’t a dead end! We can stop people from getting sick and treat it effectively if we can diagnose it at its early stage. To facilitate this efficient diagnosis, recent advancements in have enabled the rapid analysis of enormous samples, surpassing human capability^12,13^. For instance, ML, a type of AI, can reach up to 90% accuracy for various disease detection. Because of this, the majority of medical imaging analysis builds computer-aided diagnosis systems using a combination of DL and ML techniques^14,15^.

The primary objective of this research is to develop and evaluate advanced CNN architectures to enhance the accuracy, efficiency, and reliability of malaria parasite diagnosis. Specifically, it aims to design, implement, and compare the performance of three customized CNN models: PCNN, SPCNN, and SFPCNN. These models are evaluated using key metrics, including precision, recall, F1 score, accuracy, and AUC, highlighting the SPCNN model as the most effective approach. Furthermore, the research benchmarks SPCNN’s performance against widely used TL algorithms, demonstrating its superiority across evaluation measures. To provide valuable insights into decision-making processes, the interpretability of the proposed models is examined through feature activation maps, Grad-CAM, and SHAP visualizations.

The primary contributions of this research include:

- The study introduces three CNN architectures: Parallel Convolutional Neural Networks (PCNN), Soft Attention Parallel Convolutional Neural Networks (SPCNN), and Soft Attention After Functional Block Parallel Convolutional Neural Networks (SFPCNN) for malaria diagnosis.
- Sequential Preprocessing techniques like dilation, CLAHE, and normalization contribute to better feature extraction, improving the classifier’s overall performance.
- Integrating soft attention mechanisms alongside parallel convolutional architectures in the proposed SPCNN model enhances computational efficiency, performance, and feature-capturing interpretability.
- The study provides an extensive comparison with SOTA TL models, highlighting the robustness and efficiency of the proposed methods.
- External validation on multiple datasets ensures the model’s generalization capabilities, while classification extended to malaria species and stages provides a comprehensive assessment of its robustness and applicability across various scenarios.

This research document is structured in the following manner: Sect. 2 provides an in-depth analysis of relevant literature, detailing significant studies and progress in the field of malaria parasite classification through the application of DL methodologies. Section 3 presents the suggested framework, detailing the dataset collection process, providing a description of the dataset, and outlining the architectural design of the models utilized. This section also covers the classification metrics and loss functions utilized during the training process. Section 4 presents an analysis and comparison of results, offering a comprehensive evaluation of the models’ performance in comparison to other leading approaches in the field. Section 5 finalizes the article by summarizing the findings and contributions while also highlighting the practical implications and potential future directions of the research.

### Related work

Globally, several research initiatives have been put out to use data-driven DL and ML techniques for clinical settlement^16^. This study analyzed contemporary applications for artificial AI which are crucial for the healthcare industry, with a specific focus on the detection and classification of malaria parasites.

Table 1 shows a comparison of the previous study on Malaria diseases, showing the variety of datasets, methods, and architectures used by different researchers. A model based on VGG architecture was presented by Chakradeo et al.^10^and its efficacy in identifying cells infected with malaria was evaluated. The development of this model with a comparatively limited number of layers, according to the authors, makes it stand out. Its performance was validated using a rigorous five-fold cross-validation method, which produced the best accuracy ever recorded at 98.57%. Alnussairi et al.^12^ developed pre-trained CNN-based DL algorithms for the identification of organisms in samples of blood stained with Giemsa. The application of transfer learning has enhanced the performance of CNNs on small datasets. This study utilized three previously trained CNN frameworks: ResNet50, MobileNetV2, and VGG19. The NIH Malaria Dataset was used to assess the experimental result. In a separate study conducted by Liang et al.^17^, a CNN of 16 layers was suggested for enhancing the efficiency of malaria diagnosis, which has conventionally depended on the manually performed testing of blood smears using a microscope. Being evaluated on 27,578 images using ten-fold cross-validation, it obtained 97.37% accuracy. The model beat a transfer learning strategy by 91.99% on criteria such as sensitivity, specificity, F1 score, and Matthews correlation coefficient. This displays CNN’s ability to recognize contaminated cells. The study identifies CNNs as a more effective technique for malaria diagnosis. In another study, the researchers Vijayalakshmi et al.^18^, a DL approach combining transfer learning with a SVM was suggested for the identification of Plasmodium falciparum malaria from the images of microscope. The hybrid framework used pre-trained VGG layers for feature extraction and replaced later layers with an SVM classifier. Evaluated on NIH malaria dataset, it achieved 93.1% accuracy, surpassing traditional CNNs in precision, sensitivity, F1 score, and specificity. A DB network was employed to classify the parasites of malaria in human peripheral blood sample images, by the study of Bibin et al.^19^. The network trained on 4100 images used hue and texture features with contrastive divergence for pre-training and backpropagation for fine-tuning. The model, with 484 input nodes, four hidden layers, and two output nodes, achieved an F-score of 89.66%, specificity of 95.92%, and sensitivity of 97.60%. It outperformed other methods in malaria detection. The study conducted by Dong et al.^20^ concentrated on applying DL to automatically identify cells that are infected with malaria. Four pathologists labeled a dataset of red blood cells using complete slide photos of thin blood smears. While the SVM approach only managed a 92% success rate, three popular CNNs, such as- AlexNet, LeNet, and GoogLeNet—achieved 95% or higher classification accuracy. These DL models also require less human intervention because they automatically learn features from input data, improving the automation of malaria detection.

**Table 1:** Summary of some existing works on Malaria disease.

Reference	Research Activity	Dataset (with class)	Proposed Model	Performance	Limitation	XAI
Chakradeo et al.^10^	Malaria detection	NIH Malaria Dataset (Binary class)	Shallow VGG-based model with 6 convolutional layers	Accuracy: 98.22%	Struggles to distinguish between infected and contaminated cells Requires more precise region analysis No XAI is used	––
Alnussairi et al.^12^	Malaria detection	NIH Malaria Dataset (Binary class)	Deep CNN (VGG19, ResNet50, MobileNetV2) with TF	Accuracy: 100%	Limited to small datasets Potential overfitting on small datasets (despite high accuracy) Dependency on pre-trained models (may not generalize well to new or unseen malaria strains) No XAI is used	––
Liang et al.^17^	Classifying malaria-infected cells from thin blood smears	NIH Malaria Dataset (Binary class)	Custom 16-layer CNN	Accuracy: 97.37%	Overfitting risks Limited generalization to diverse datasets No XAI is used	––
Vijayalakshmi et al.^18^,	Identification of infected P. falciparum malaria parasite	NIH Malaria Dataset (Binary class)	VGG19-SVM (TF + SVM)	Accuracy: 93.1%	Increased Model complexity Requires extensive preprocessing and domain-specific expertise No XAI is used	––
Bibin et al.^19^	Malaria parasite detection in blood smear images	NIH Malaria Dataset (Binary class)	DBN with 484–600-600–600-600–2 architecture, using concatenated color and texture features	F-score: 89.66%	High computational cost Limited generalization Requires optimal DBN hyperparameter tuning for consistent results No XAI is used	––
Dong et al.^20^	Identification of malaria-infected cells	PEIR-VM repository (UAB) with malaria-infected and non-infected RBCs	LeNet, AlexNet, GoogLeNet (CNNs)	Accuracy: 95%	High computational cost Limited device generalization Inability to identify specific malaria species No XAI is used	––
Madhu et al.^21^	Malaria classification	NIH Malaria Dataset (Binary class)	Capsule Network with Modified Routing Algorithm	Accuracy: 98.82%	Dependence on hand-crafted features Sensitivity to loss parameter tuning Limited generalization across different imaging conditions Lack of species and stage identification High computational complexity Potential overfitting No XAI is used	––
Ha et al.^22^	Apicomplexan parasite recognition	Mendeley Microdata of Parasite Microbiology Dataset	Semi-Supervised Graph Learning (SSGL) with CNN feature embedding and graph-based learning	Accuracy: 91.75%	Dependence on a small labeled dataset Limited generalization across different types of apicomplexan parasites Potential limitations in capturing complex correlations among cells No XAI is used	––
Ramirez et al.^23^,	Classification of malaria-infected RBCs	NIH Malaria Dataset (Binary class)	Conv-LSTM & Bi-LSTM	Accuracy: 99.89%	Dependency on labeled datasets Lack of model generalization to unseen data No XAI is used	––
Umana et al.^26^	Malaria detection in RBC images	NIH Malaria Dataset (Binary class)	DepthResInceptNet	Accuracy: 94.2%	Model complexity may affect real-time performance Requires significant computational power Limited to the dataset’s generalizability	Grad-CAM
Mridha et al.^27^	Malaria detection	NIH Malaria Dataset (Binary class of blood smear images)	ResNet50, CNN, MobileNet	Accuracy: 96.0%	High computational requirements for training Dependent on labeled datasets Lack of model generalization	Grad-CAM
Agrawal et al.^28^	Malaria detection	NIH Malaria Dataset (Binary class of blood smear images)	FixMatch (Semi-supervised), Supervised Model	Accuracy: 97%	Overfitting in limited labeled data Dependency on high-quality labeled data Challenges in data diversity for broader generalization	SHAP, Grad-CAM
Islam et al.^29^	Malaria detection	NIH Malaria Dataset (Binary class of blood smear images)	Multi-headed Attention-based Transformer	Accuracy: 96.41%	Exclusion of segmentation step (used pre-segmented RBC images, limiting real-world applicability) Non-consideration of parasite life stages	Grad-CAM
Goni et al.^30^	Malaria detection	NIH Malaria Dataset (Binary class of blood smear images)	Customized Lightweight CNN	Accuracy: 99.45%	Limited generalizability Focused on a small number of model parameters for faster processing	SHAP
Devi et al.^24^	Malaria infected erythrocyte classification	NIH Malaria Dataset (Binary class of blood smear images)	Hybrid classifier (SVM, k-NN, Naive Bayes) with optimal feature set	Accuracy: 98.5%	Feature extraction complexity High dependency on optimal feature selection Limited generalization Computational cost of hybrid approach No XAI is used	––
Raihan et al.^25^	Malaria detection	NIH Malaria Dataset (Binary class of blood smear images)	Wavelet Packet 2D + CNN + Whale Optimization Algorithm (WOA) + XGBoost	Accuracy: 94.78%	Complexity in feature Feature selection dependency Limited interpretability of feature interactions (relying only on SHAP for interpretation but may not capture all complex interactions) Potential overfitting on selected feature set	SHAP

Madhu et al.^21^ presented a novel method of classifying malaria that uses capsule networks rather than the more conventional CNNs. Capsule networks, as opposed to CNNs, are better at handling rotating picture fluctuations and preserving spatial information. The authors created an imperative dynamic routing technique to overcome the shortcomings of earlier methods. On test samples, the model shows an AUC of 99.03% and a specificity of 99.43%. Nevertheless, these previous studies have a drawback in that they need manually labeled samples by specialists. In contrast to conventional techniques, Ha et al.^22^ presented a novel strategy termed the SSGL framework to use DL to automate the identification of apicomplexan parasites. CNNs are combined in SSGL to lessen the requirement for labeled data. With only 20% of labeled data, the approach achieves good accuracy (91.75%), AUC (91.83%), sensitivity (91.75%), and specificity (97.25%). A recent study by Ramirez et al.^23^, utilized DL to automate the identification of malaria-infected RBCs, traditionally reliant on microscopy. They proposed two Convolutional RNN architectures: Convolutional LSTM and Convolutional BiLSTM, achieving 99.89% accuracy on a public malaria dataset. These models demonstrated high effectiveness with minimal preprocessing. Their work underscores the potential of CRNNs to improve malaria diagnostic methods.

In the realm of ML for malaria parasite classification, a hybrid classifier-based computer-assisted approach was suggested by Devi et al.^24^ to find out the erythrocytes infected with malaria. It makes use of a collection of 54-dimensional features that are obtained from textural and intensity-based data. The evaluation focusses on the effectiveness of various classifiers, including k-NN, SVM, and Naive Bayes, both individually and as part of an integrated classifier which combines these different methods. Applying the hybrid model and this ideal feature set, the system performs better on the clinical database, with F1-score (93.82%), sensitivity (95.86%), and accuracy (98.5%). Raihan et al.^25^ reported that XGBoost attained an accuracy of 94.78% while maintaining equally good precision and recall. The study used SHAP to analyze feature contributions, however, it did not address SHAP’s shortcomings in explaining non-linear interactions in medical imaging data. Furthermore, the lack of consideration of the practical constraints of implementing such interpretability frameworks in real-world diagnostics highlights a key gap.

Despite significant improvements in performance indicators, most of the current studies on malaria parasite categorization overlook interpretability. Umana et al.^26^ obtained 94.20% accuracy on the NIH Malaria Dataset utilizing DepthResInceptNet and Grad-CAM for model interpretation. However, they failed to solve Grad-CAM’s difficulties in capturing complicated feature hierarchies. In another study Mridha et al.^27^ showed 95.00% accuracy using MobileNet, with Grad-CAM help for prediction interpretation. However, their research falls short in addressing how Grad-CAM may struggle to pinpoint tiny traits that are critical for correct malaria diagnosis. This absence raises concerns regarding the validity of their interpretability method in clinical settings. The FixMatch model, a semi-supervised learning approach, on the NIH Malaria Dataset, achieved 96.00% accuracy, 93.80% precision, and 97.10% recall, using both Grad-CAM and SHAP for explainability implemented by Agrawal et al.^28^. Rajab et al. used XAI approaches, notably SHAP, to improve interpretability in severe malaria forecasts. While methods like Random Forest and EBM highlight the relevance of certain features, XGBoost’s intricacy makes it difficult to grasp. SHAP emphasizes feature significance but struggles to capture complex feature interactions in XGBoost. This yields less accurate insights into the decision-making process. Transformers, used by Islam et al.^29^, attained 96.41% accuracy with 99.08% precision and recall, employing Grad-CAM for model interpretation. Their findings demonstrate strong performance, with Grad-CAM providing insights into the model’s decision-making process. A study by Goni et al.^30^, introduces a lightweight CNN for rapid malaria detection from RBC images, achieving 99.45% accuracy, 99.75% precision, 99.17% recall, and 99.46% F1-score. With only 0.17 million parameters, it outperforms or matches traditional transfer-learning models and state-of-the-art methods. SHAP is used to explain the model’s decisions, enhancing its interpretability. The proposed method demonstrates high efficiency and performance for malaria detection.

The mentioned studies demonstrate the effectiveness of DL and ML techniques in malaria diagnosis, but several limitations persist. Most models are computationally expensive, making implementation in resource-constrained environments impracticable. They frequently lack generalizability since evaluations are limited to internal datasets, reducing real-world applicability. Robustness against noisy or low-quality inputs is not adequately addressed, creating concerns regarding dependability in clinical settings. While most research didn’t incorporate XAI approaches, a few studies explored SHAP and Grad-CAM, as these methods are particularly suitable for interpreting model decisions in healthcare applications. However, there is a notable limitation in comparative studies related to interpretability, as few efforts have been made to systematically evaluate and contrast the performance of different XAI methods in terms of clarity, reliability, and clinical relevance. This limits the ability to identify the most effective techniques for aiding practitioners in understanding model outputs. Additionally, comprehensive evaluation of training efficiency is still lacking, as many utilize big, complicated designs that require substantial computing resources. The effects of hyperparameter adjustment and data imbalance on performance are rarely studied. Furthermore, challenges in dealing with various data distributions and adaptive learning for changing parasite features remain unexplored. Addressing these problems will enhance the usability and efficacy of DL and ML-based malaria diagnostic methods.

### Methodology

#### Proposed framework

**Fig. 1:**
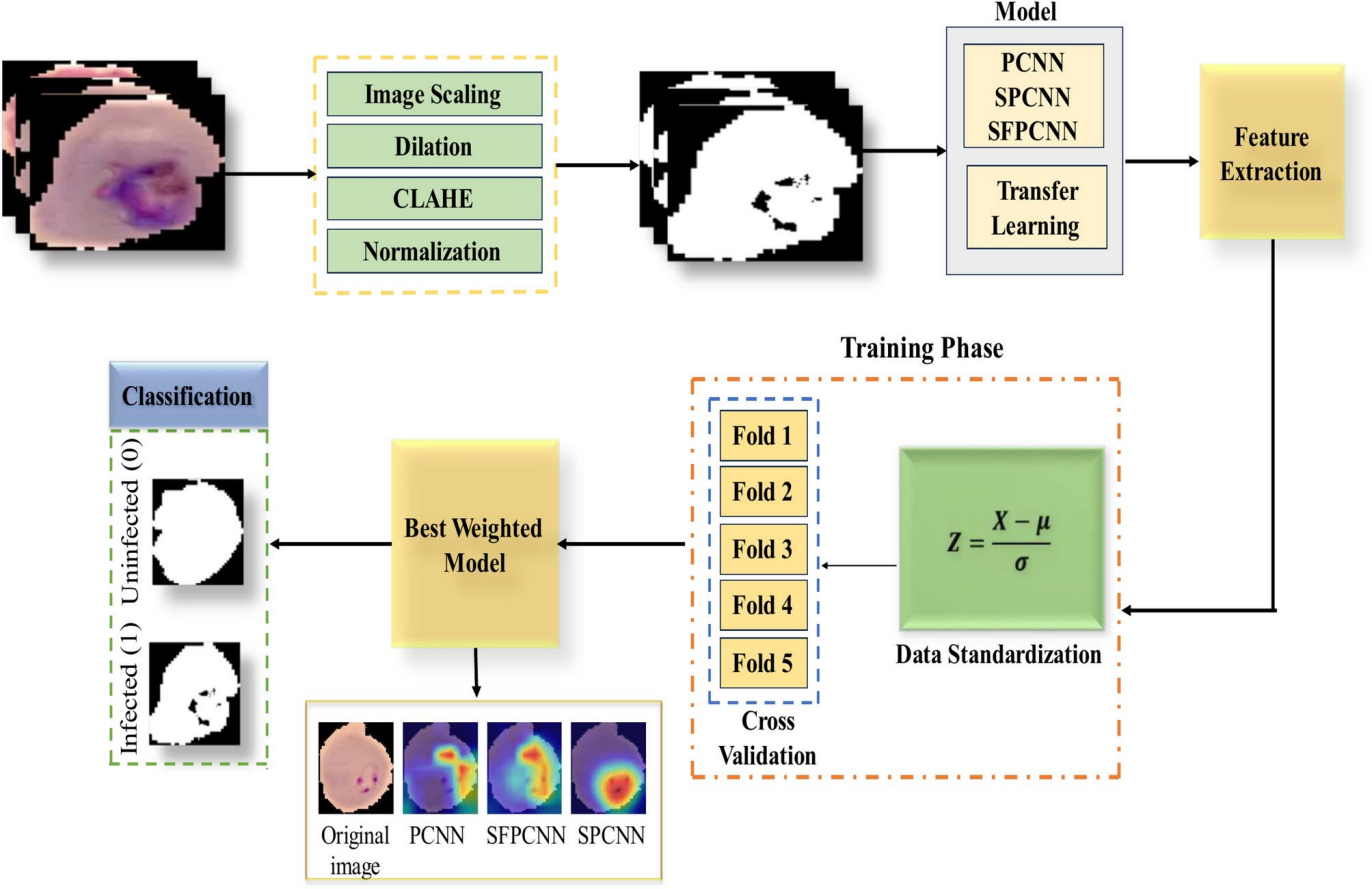
The framework proposed for classifying malaria parasites from RBC smear samples.

A comprehensive framework for classifying malaria parasites from RBC smears, including cases from healthy people, is shown in Fig. 1. The initial stage is to gather a large dataset of pictures of both diseased and healthy people via the National Library of Medicine. During the preprocessing stage, many methods are used to improve the quality of the images and get the data ready for investigation. By standardizing the proportions of the images, image resizing ensures uniformity and qualifies them to be utilized as input in neural network models. This phase is essential for preserving consistency throughout the dataset and improving CNN architecture performance. The images’ contrast is enhanced with the application of CLAHE. CLAHE functions by enhancing local contrast, which proves particularly beneficial for highlighting the subtle details in RBC images. By highlighting significant elements that may not be as obvious in the original images, this technique improves feature extraction and categorization. Another essential preprocessing step is normalization. Scaling the values of the pixels to a common span, typically between 0 and 1, enhances the training convergence of the neural network. By ensuring that the data distributions of each image are consistent, normalization can greatly enhance the models’ performance and training stability^31^. The framework uses several CNN architectures, including SFPCNN, SPCNN, and PCNN.

To ensure uniformity and comparability of extracted features during training, feature standardization is performed using the formula,

1 $${\varvec{z}}=\frac{{\varvec{x}}-{\varvec{\mu}}}{{\varvec{\sigma}}}$$

where $z$ represents the standardized feature, $x$ is the original feature value, $\mu$ is the mean of the feature values, and $\sigma$ is the standard deviation of the feature values^32^. Standardizing features ensures that they have a mean of 0 and a standard deviation of 1, which prevents certain features with larger magnitudes from disproportionately influencing the model training process. This step enhances the stability and convergence of the learning algorithms and ensures that all features contribute equally during model training.

These structures intend to capture the spatial and contextual characteristics of the RBC images well. By specifically designed to handle the intricate patterns present in malaria-infected cells, the customized CNN model improves the accuracy of detection. TL models such as VGG16, ResNet152, MobileNetV3Small, EfficientNetB6, EfficientNetB7, and DenseNet201 are also used to classify malaria parasites. The outcomes are compared with our suggested tailored CNN approaches. These algorithms efficiently identify characteristics in the malaria photos with little training time because they use pre-trained weights from big image datasets.

### Dataset collection

Using Giemsa-stained thin samples of blood collected at Chittagong Medical College Hospital in Bangladesh, the framework was evaluated on a dataset including 150 P. falciparum parasitized and 50 unaffected patients.

**Fig. 2:**
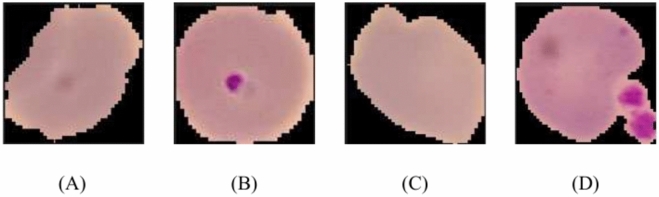
Depictions of blood smear cases featuring (A) accurately identified uninfected cells, (B) accurately identified parasitized cells, (C) mislabeled infected cells, and (D) mislabeled uninfected cells.

The Lister Hill National Centre for Biomedical Communication, a branch of the National Library of Medicine, facilitates access to the dataset^33^. A professional slide reader at Thailand’s Mahidol Oxford Tropical Medicine Research Unit (Bangkok) manually labeled each image. There are 27,558 photos in total, with an equal number of parasitized and healthy classes^34^. In this data, a parasitized sample contains plasmodium, whereas uninfected samples lack plasmodium but may contain artifacts or other contaminants. Fuhad et al.^35^ encountered some mislabeling on the dataset where some uninfected samples were labeled as parasitized, and some parasitized instances were labeled as uninfected. To fixed this issue the dataset was further labeled by an expert and falsely annotated data was put aside. After the reannotation, the dataset was reduced to 26,161 from 27,558. Throughout this rectification process, they removed 647 falsely parasitized samples, and the current amount of parasitized sample stands at 13,132. The total true uninfected data was found 13,029 by discarding the 750 samples those was named as falsely uninfected. Some random examples from the dataset are depicted in Fig. 2.

### Dataset Pre-processing

An image’s preprocessing quality has a major impact on how well a classification model performs. We begin by scaling the image to 32 × 32 pixels to do this. Next, we use a set of well-known methods, such as CLAHE and Dilation, to improve our image preparation approach. Morphological dilation is an effective way to combine broken portions of an image so that its constituent pieces are represented more coherently and continuously. This method enhances the interpretability of the processed image by emphasizing particular structures or features in addition to improving the forms of entities.

**Fig. 3:**
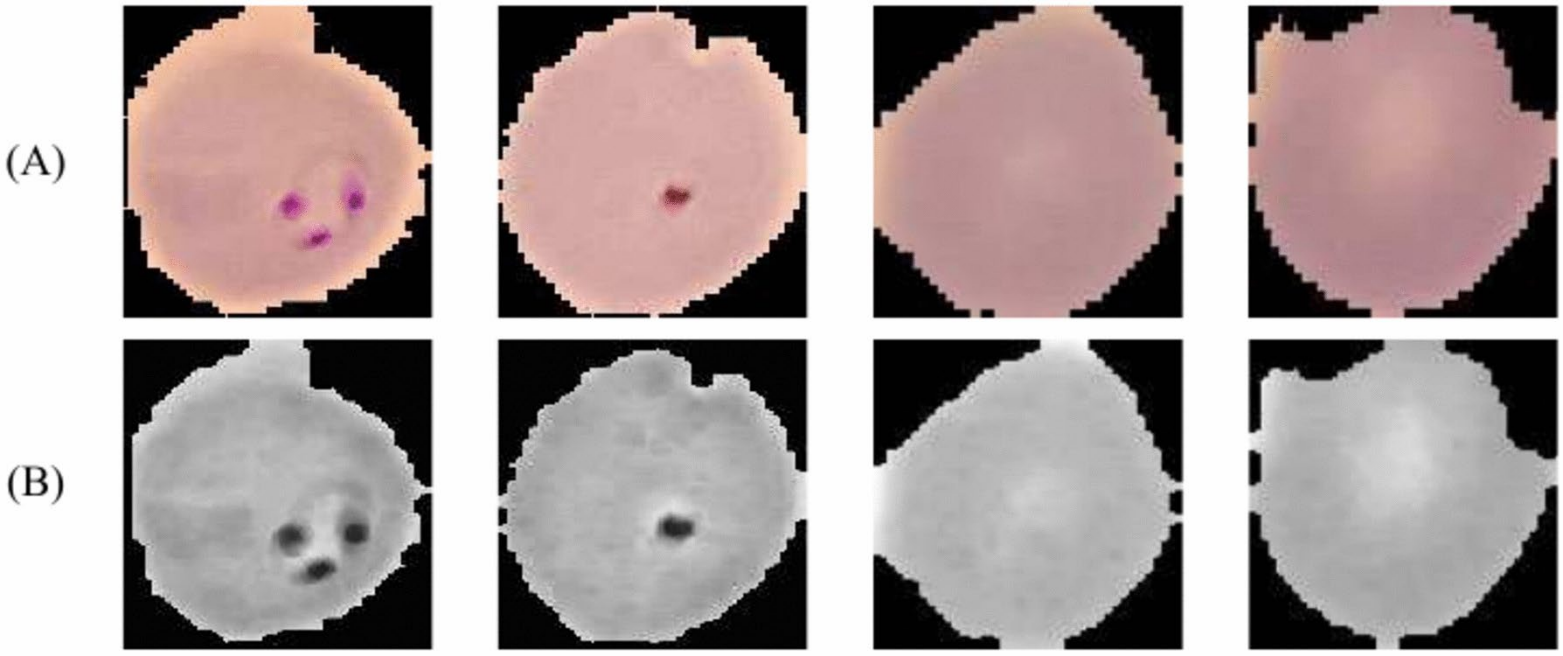
RBC blood smear segments (**A**) before preprocessing and (**B**) after the application of Dilation, CLAHE, and Normalization techniques.

Dilation also successfully minimizes undesired artifacts and noise, which makes it a crucial component of our hybrid preprocessing method^36^. By reducing the noise amplification, it improves the image’s local contrast, particularly in regions that could have poor contrast. Through small-scale histogram equalization, CLAHE guarantees enhanced overall contrast without oversaturating the brighter areas of the image^37^. As a result, the image is improved more consistently, which can greatly help with accurate feature recognition and categorization. When combined, Dilation and CLAHE provide a strong preprocessing framework that improves feature visibility, lowers noise, and improves image quality—all of which contribute to improved model performance. Furthermore, dividing the pixel values by 255 to normalize the images makes the analysis that follows much easier, and it also drastically lowers the computing complexity of the model^38^. Hematologists can use this method with confidence to increase the detection accuracy of malaria. By enhancing the readability of human blood cells, the CLAHE and Dilation procedures can be applied together to provide a more precise detection of malaria. CLAHE improves local contrast, drawing attention to minute irregularities, and Dilation efficiently reduces noise without sacrificing important aspects of the image. The samples are shown in Fig. 3 both before and after preprocessing. The images are prepared for feeding into our classification models, which include the PCNN, SPCNN, and SFPCNN, after undergoing extensive preprocessing.

### Parallel convolutional neural network (PCNN)

**Fig. 4:**
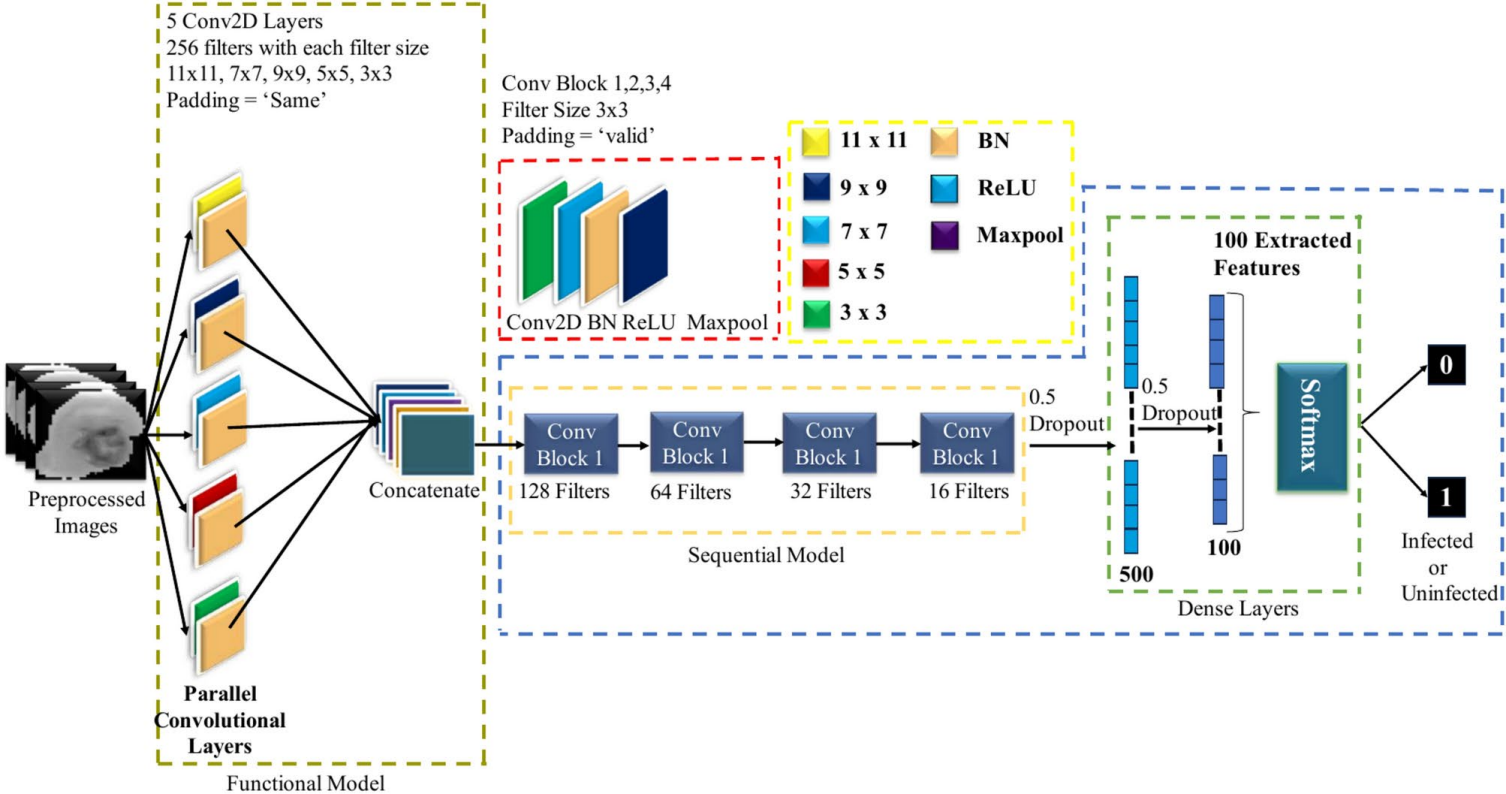
Detailed block diagram of PCNN architecture.

Convolutional layers with two-dimensional kernels applied along the time axis are used in PCNNs to handle dynamic energy characteristics while preserving information from each channel^39^. PCNN is used in malaria parasite classification to increase the precision of parasite detection in red blood cell pictures. The PCNN design employs a number of parallel convolutional layers, with the goal of extracting different types of spatial information from the pictures being used at the same time. A more thorough and reliable representation of the malaria parasites is made possible by the network’s ability to extract a wide range of features thanks to this parallel processing. The Fig. 4 demonstrates the PCNN architecture, illustrating how parallel layers work together to analyze various features of the input images simultaneously. The PCNN may incorporate different viewpoints on the input data by integrating the outputs of these parallel layers, producing improved feature maps. To enhance the data processing and identify the most relevant features for classification, these feature maps are enhanced with pooling and fully connected layers. In order to make the model faster and more reliable, batch normalization (BN) was used. In training process, Rectified Linear Unit (ReLU) activation functions were incorporated into all convolutional layers (CLs) to introduce nonlinearity while ensuring consistency. Dropout layers with a 0.5 probability were used to mitigate overfitting and expedite training. These layers randomly deactivate 50% of the network’s nodes. One dropout layer was added after the first fully connected (FC) layer and another one was added after the last two convolutional layers. The loss function selected for the ADAM optimizer was sparse categorical cross-entropy, and the framework underwent training for 100 epochs with a learning rate set at 0.001%.

The model’s performance was evaluated by extracting 100 distinct features from the final FC layer through a process of trial and error. Table 2 presents an extensive overview of the PCNN model, offering an in-depth summary of its architecture and structural components.

**Table 2:** Detailed summary of the PCNN Model [Where, C2D = Conv2D, BN = Batch Normalization, Act = Activation, MP = Max-Pooling, DO = Dropout, F = Flatten and D = Dense].

Layer Type	Output Shape	Parameters
Functional	(None, 32, 32, 1280)	220,160
C2D	(None, 32, 32, 128)	1,474,688
BN	(None, 32, 32, 128)	512
Act	(None, 32, 32, 128)	0
MP	(None, 16, 16, 128)	0
C2D	(None, 16, 16, 64)	73,792
BN	(None, 16, 16, 64)	256
Act	(None, 16, 16, 64)	0
MP	(None, 8, 8, 64)	0
C2D	(None, 8, 8, 32)	18,464
BN	(None, 8, 8, 32)	128
Act	(None, 8, 8, 32)	0
MP	(None, 4, 4, 32)	0
C2D	(None, 4, 4, 16)	4624
BN	(None, 4, 4, 16)	64
Act	(None, 4, 4, 16)	0
MP	(None, 2, 2, 16)	0
DO	(None, 2, 2, 16)	0
F	(None, 64)	0
D	(None, 500)	32,500
BN	(None, 500)	2000
DO	(None, 500)	0
D	(None, 100)	50,100
BN	(None, 100)	400
DO	(None, 100)	0
D	(None, 2)	202

### Soft attention parallel convolutional neural network (SPCNN)

**Fig. 5:**
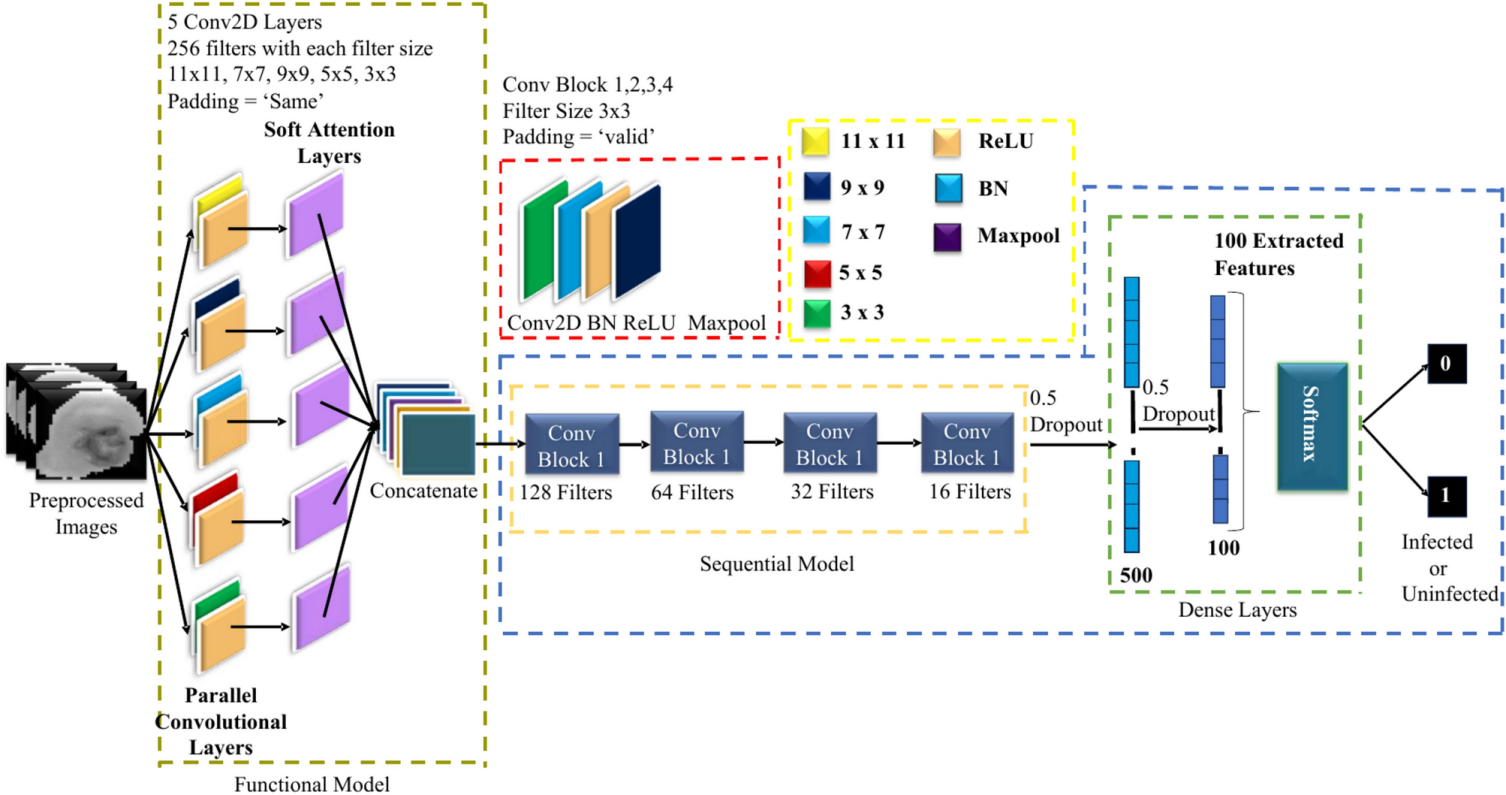
Block diagram of proposed SPCNN architecture.

Our primary goal was to develop a Customized CNN model that could accurately identify key characteristics while staying compact and helpful for diagnosing malaria parasites^40^. To accomplish this aim, we created a simplified SPCNN model that balances layer complexity and parameter limitations. The architecture of this model, which successfully recovers unique characteristics from RBC-segmented pictures, is shown in Fig. 5. To render the CNN method more approachable than Transfer Learning, we used an advanced strategy. The model is made up of eight CL and two fully connected layers (FC), which were precisely balanced during the construction process. A single CL would be devoid of important subtleties, while five CLs in succession would add needless nuance and complication. With the help of a lot of testing, we concurrently built the first five CLs using concatenation. To guarantee thorough feature extraction and precise classification, 256 kernels in sizes ranging from 11 × 11 to 3 × 3 were used in each of these layers. These first layers were padded consistently to enable full data extraction from all locations, including the border areas. Feature maps were produced by the parallel convolutional layers, which were then combined and subjected to extra processing by a sequential convolutional layer. We used 3 × 3 kernels with appropriate padding in the batch normalization (BN) and max-pooling layers, which we added after the first four convolutional layers, to increase accuracy. For these layers, we determined that the ideal filter sizes were 128, 64, 32, and 16. Table 3 presents a comprehensive overview of the model, encompassing the parameters of every layer. Batch normalization (BN) was incorporated to enhance both the speed and reliability of the model. To maintain consistency and nonlinearity, ReLU activation functions were applied across all CLs. Dropout layers, with a 50% probability, were added to reduce overfitting and accelerate training by randomly deactivating half of the network’s nodes. Specifically, dropout layers were placed after the first FC layer and following the final two convolutional layers. The model was trained using the ADAM optimizer with a learning rate of 0.001% for 100 epochs, and sparse categorical cross-entropy was employed as the loss function. For performance evaluation, 100 unique features were selected from the final FC layer using trial and error.

**Table 3:** Summary of the SPCNN Architecture [Where, C2D = Conv2D, BN = Batch Normalization, Act = Activation, MP = Max-Pooling, DO = Dropout, F = Flatten and D = Dense].

Layer Type	Output Shape	Parameters
Functional	(None, 32, 32, 1280)	549,120
C2D	(None, 32, 32, 128)	1,474,688
BN	(None, 32, 32, 128)	512
Act	(None, 32, 32, 128)	0
MP	(None, 16, 16, 128)	0
C2D	(None, 16, 16, 64)	73,792
BN	(None, 16, 16, 64)	256
Act	(None, 16, 16, 64)	0
MP	(None, 8, 8, 64)	0
C2D	(None, 8, 8, 32)	18,464
BN	(None, 8, 8, 32)	128
Act	(None, 8, 8, 32)	0
MP	(None, 4, 4, 32)	0
C2D	(None, 4, 4, 16)	4624
BN	(None, 4, 4, 16)	64
Act	(None, 4, 4, 16)	0
MP	(None, 2, 2, 16)	0
DO	(None, 2, 2, 16)	0
F	(None, 64)	0
D	(None, 500)	32,500
BN	(None, 500)	2000
DO	(None, 500)	0
D	(None, 100)	50,100
BN	(None, 100)	400
DO	(None, 100)	0
D	(None, 2)	202

### Soft attention after functional block parallel convolutional neural network (SFPCNN).

The location of the soft attention mechanism is where SFPCNN and SPCNN diverge most. After the parallel convolutional layers have retrieved features and concatenated them, soft attention is used in SFPCNN. This method can help capture intricate patterns globally by giving the network the ability to evaluate a whole view of all extracted characteristics and apply varying weights to highlight the most significant ones. Dividing the feature extraction and attention steps streamlines the model and facilitates training.

**Fig. 6:**
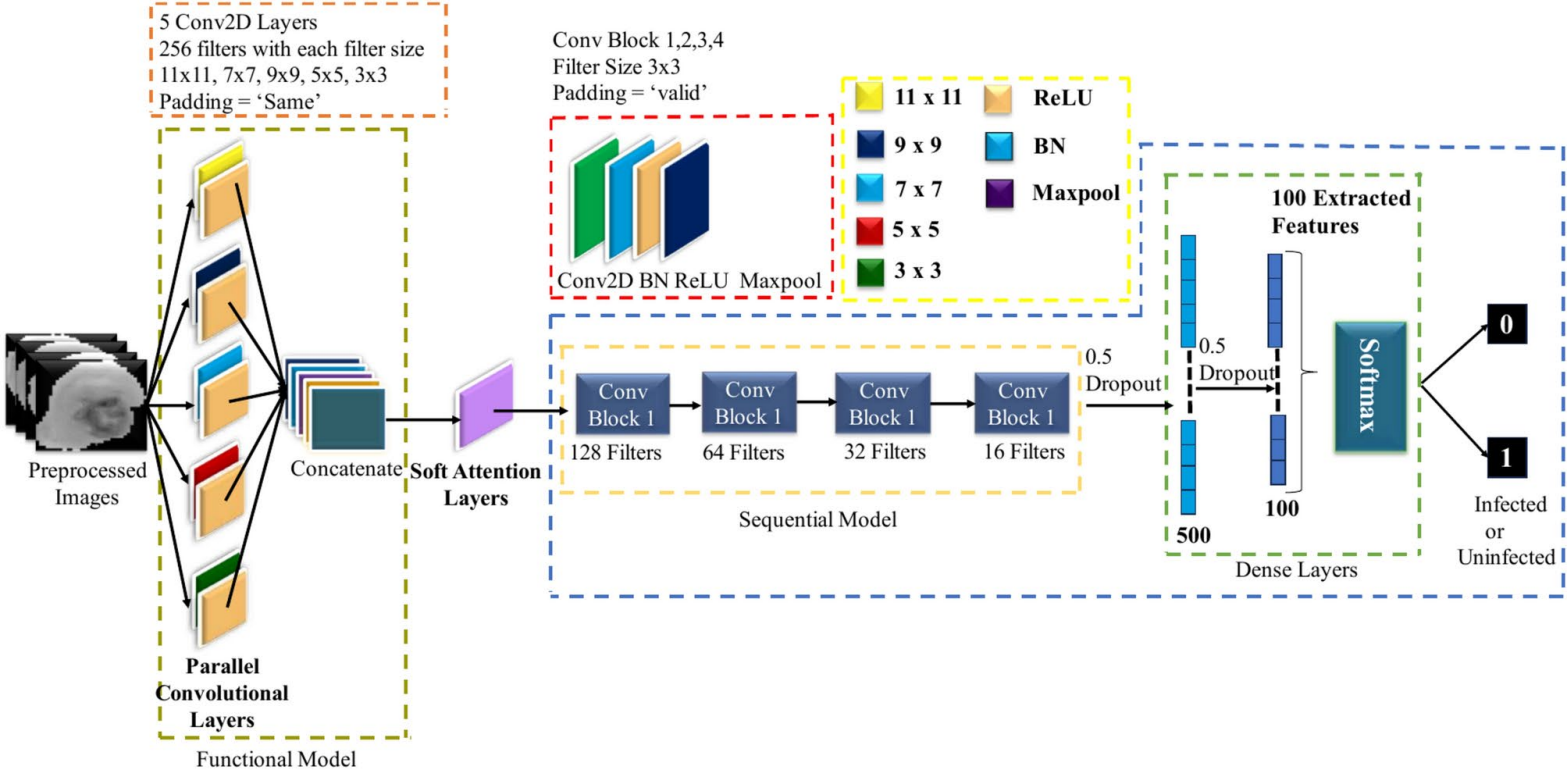
Block diagram of SFPCNN architecture for feature extraction.

In Fig. 6 describes the architecture of SFPCNN, highlighting how soft attention is applied after feature extraction, ensuring that the network focuses on the most crucial features before classification. In contrast, SPCNN incorporates the soft attention mechanism into every functional block while the features are being extracted. Like SPCNN the SFPCNN architecture was designed with 256 kernels ranging in size from 11 × 11 to 3 × 3, which allowed for thorough feature extraction. Consistent padding was used on these layers to obtain information from all regions of the image, including borders. The parallel convolutional layers generated feature maps, which were then combined and refined using a sequential convolutional layer to improve pattern recognition. To improve accuracy, batch normalization and max- pooling layers were added after the first four convolutional layers. Batch normalization improved both the model’s speed and stability, while ReLU activation was used consistently across all convolutional layers to maintain nonlinearity. To reduce overfitting, dropout layers with a rate of 0.5 were added after the first fully connected layer and the last two convolutional layers. The model was optimized using the ADAM optimizer with a 0.001% learning rate over 100 epochs, with sparse categorical cross-entropy as the loss function. Finally, 100 different characteristics were chosen from the final fully connected layer through trial and error to assess the model’s performance. Table 4 offers a summary of the SFPCNN model, detailing its architecture and key design components.

**Table 4:** Soft attention after functional block parallel convolutional neural network (SFPCNN) model summary [Where, C2D = Conv2D, BN = Batch Normalization, Act = Activation, MP = Max-Pooling, DO = Dropout, F = Flatten and D = Dense].

Layer type	Output shape	Parameters
Functional	(None, 32, 32, 1280)	1,859,840
C2D	(None, 32, 32, 128)	1,474,688
BN	(None, 32, 32, 128)	512
Act	(None, 32, 32, 128)	0
MP	(None, 16, 16, 128)	0
C2D	(None, 16, 16, 64)	73,792
BN	(None, 16, 16, 64)	256
Act	(None, 16, 16, 64)	0
MP	(None, 8, 8, 64)	0
C2D	(None, 8, 8, 32)	18,464
BN	(None, 8, 8, 32)	128
Act	(None, 8, 8, 32)	0
MP	(None, 4, 4, 32)	0
C2D	(None, 4, 4, 16)	4624
BN	(None, 4, 4, 16)	64
Act	(None, 4, 4, 16)	0
MP	(None, 2, 2, 16)	0
DO	(None, 2, 2, 16)	0
F	(None, 64)	0
D	(None, 500)	32,500
BN	(None, 500)	2000
DO	(None, 500)	0
D	(None, 100)	50,100
BN	(None, 100)	400
DO	(None, 100)	0
D	(None, 2)	202

### Soft attention mechanism

The soft and spatial attention mechanisms aim to highlight essential aspects of input data but differ in their focus and application. Soft attention assigns continuous attention weights to all input elements, enabling a global relevance evaluation across sequences or feature sets, including those extracted from images. This mechanism is widely used in image-based applications, like image classification, by emphasizing the most relevant feature regions. In contrast, spatial attention is explicitly tailored for computer vision tasks. It focuses on localized areas within an image, targeting the spatial dimensions of 2D feature maps to identify the most significant regions for tasks like object detection or segmentation^41^. The primary distinction is that while soft attention evaluates relevance globally across the entire input, spatial attention highlights critical spatial areas within visual data.

**Fig. 7:**
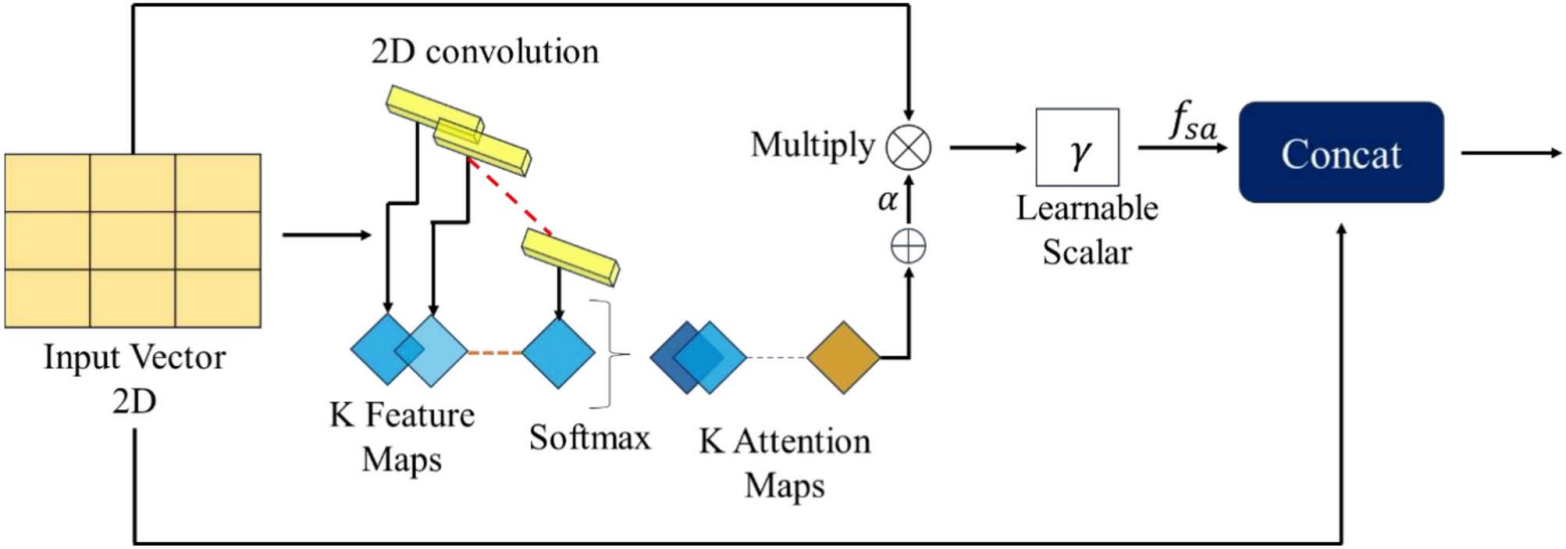
Architecture of soft attention mechanism.

Soft attention mechanisms portrayed in Fig. 7 have grown in popularity as a useful method for improving CNN’s performance on various tasks, including the categorization of medical images. This method enables the network to concentrate on significant areas of the picture, which is very helpful for locating malaria parasites in blood smear pictures. Neural networks’ attention processes are modeled after the human visual attention system, which enables models to process information while focusing on particular segments of an input^42^. The goal is to improve the model’s capacity to focus on pertinent characteristics while disregarding less significant data by allocating varying weights to various input components. This mechanism is particularly useful when dealing with data where only certain regions or components are important for the task at hand, such as in image classification or natural language processing^43^. The goal is to guide the network to focus more on informative areas, such as potential malaria-infected cells in a blood smear, and less on background or irrelevant parts of the image. The process starts by taking an input feature map t $\in {\mathbb{R}}^{H\times W}$, Where H and W denote the feature map’s height and width, respectively. The feature tensor t is processed through a 2D convolutional filters. ${W}_{k}\in {\mathbb{R}}^{H\times W\times K}$ to produce multiple attention maps ${a}_{k},$ where k is the number of 2D weights.

2 $${{\varvec{a}}}_{{\varvec{k}}}={{\varvec{W}}}_{{\varvec{k}}}\boldsymbol{*}{\varvec{t}}$$

The softmax function is then used to normalize these attention maps, giving probabilistic weights ${\alpha }_{k}$ to each spatial position (i, j) in the feature map to highlight the most significant areas.

3 $${\boldsymbol{\alpha }}_{{\varvec{k}}}({\varvec{i}},{\varvec{j}})=\frac{{{\varvec{e}}}^{{{\varvec{a}}}_{{\varvec{k}}}({\varvec{i}},{\varvec{j}})}}{{\sum }_{{\varvec{k}}=1}^{{\varvec{K}}}{{\varvec{e}}}^{{{\varvec{a}}}_{{\varvec{k}}}({\varvec{i}},{\varvec{j}})}}$$

Furthermore, the several attention maps are combined to create a single attention map α, as seen below:

4 $$\boldsymbol{\alpha }={\sum }_{{\varvec{k}}=1}^{{\varvec{K}}}{{\varvec{a}}}_{{\varvec{k}}}$$

Then, using element-wise multiplication as a gating mechanism, this attention map, α, scales the original feature map, *t*.

5 $${{\varvec{f}}}_{{\varvec{s}}{\varvec{a}}}=\boldsymbol{ }{\varvec{\gamma}}(\boldsymbol{\alpha }\odot \mathbf{t})$$

Here, $\gamma$ is a learnable scalar that is initialized to a low value of 0.01, so the network can dynamically control the attention’s effect throughout training. Finally, a residual connection is used to merge the attentively scaled features (${f}_{sa}$) with the original feature map t:

6 $${{\varvec{f}}}_{{\varvec{r}}{\varvec{e}}{\varvec{s}}}={\varvec{t}}+{{\varvec{f}}}_{{\varvec{s}}{\varvec{a}}}$$

This combination increases recognition performance by preserving important original information and augmenting it with attention-refined characteristics.

Procedure of Performance Evaluation and Loss Function.

To evaluate performance, the model underwent training, validation, and testing phases, with metrics such as accuracy, precision, recall, and F1-score employed to measure its effectiveness^44^. These steps ensured a comprehensive assessment of the model’s recognition capabilities. The details of the model training setup and system specifications are presented in Table 5.

7 $$Accuracy= \frac{{T}_{P}+{T}_{N}}{{T}_{P}+{T}_{N}+{F}_{P}+{F}_{N}}$$

8 $$Precision= \frac{{T}_{P}}{{T}_{P}+{F}_{P}}$$

9 $$Recall= \frac{{T}_{P}}{{T}_{N}+{F}_{P}}$$

10 $$F1-Score= \frac{2*Precison*Recall}{Precision+Recall}$$

11 $$AUC= \frac{1}{2}\left(\frac{{T}_{P}}{{T}_{P}+{F}_{N}}+\frac{{T}_{N}}{{T}_{N}+{F}_{P}}\right)$$

here ${T}_{P}=$ True positive, ${T}_{N}=$ True Negative, ${F}_{P}=$ False Positive, and ${F}_{N}=$ False negative.

**Table 5:** Model training setup and system specifications.

Component	Specification
Framework	Python PyTorch
Processor	11th Gen Intel(R) Core (TM) i9-11,900 @ 2.50GHz
RAM	128 GB
GPU	NVIDIA GeForce RTX 3090 (24 GB)
Operating System	Windows 10 PRO (64-bit)
Loss Function	Sparse Categorical Cross-Entropy

Sparse Categorical Cross-Entropy is a loss function often used in multi-class classification situations where labels are provided as integer indices rather than one-hot encoded vectors. This loss function is very effective in DL models with a large number of target labels since it requires less memory than one-hot encoding. Sparse Categorical Cross-Entropy calculates the negative log probability of the true class label’s anticipated probability^45^.

12 $$Loss = - \mathop \sum \limits_{i = 1}^{n} y_{i} * \log \hat{y}_{i}$$

where n is the number of classes, ${y}_{i}$ is the true label for class i, and $\hat{y}_{i}$ is the predicted probability of class i.

### Hyperparameters selection

Table 6 delineates the hyperparameters evaluated and chosen for training the PCNN, SFPCNN, and SPCNN architectures. An empirical selection process identified the ideal settings, including repeated testing and validation performance. Deeper configurations ([3 × 3, 5 × 5, 7 × 7, 9 × 9, 11 × 11]) surpassed smaller layers by delivering superior accuracy without incurring overfitting. Correspondingly, filter sizes ([128, 64, 32, 16]) were selected for enhanced feature extraction and computational efficacy. A batch size of 32 optimized training velocity and model precision, while a learning rate of 0.001 facilitated steady convergence, surpassing elevated (e.g., 0.01) and diminished values (e.g., 0.0001). The efficacy of the selected dropout rate of 0.5 in mitigating overfitting was a significant achievement of the regularization technique. The Adam optimizer was chosen for its superior convergence speed relative to SGD and Nadam. At the same time, ReLU activation surpassed alternatives such as LeakyReLU and Tanh by mitigating the vanishing gradient issue. Ultimately, 100 epochs were adequate for model convergence, as evidenced by steady training and validation loss.

**Table 6:** Experimental hyperparameters summary for training PCNN, SFPCNN, and SPCNN.

Hyperparameter	Explored Values	Selected Value	Justification
Convolutional Layers Sizes (Functional model)	[3 × 3, 5 × 5, 7 × 7], [3 × 3, 5 × 5, 7 × 7, 9 × 9], [3 × 3, 5 × 5, 7 × 7, 9 × 9, 11 × 11], [3 × 3, 3 × 3, 3 × 3], [5 × 5, 5 × 5, 5 × 5], [3 × 3, 3 × 3, 5 × 5, 5 × 5, 7 × 7]	[3 × 3, 5 × 5, 7 × 7, 9 × 9, 11 × 11]	Optimal for achieving high accuracy without overfitting
Filter Sizes (Sequential model)	[128,64,32,16,8], [128,64,32,16], [128,64,32], [256,128,64,32,16,8], [256,128,64,32,16]	[128,64,32,16]	Ensured best feature extraction for image inputs
Batch Size	{16, 32, 64, 128}	32	Balanced training speed and model accuracy
Learning Rate	{0.0001, 0.001, 0.01, 0.1}	0.001	Selected based on best performance during validation
Dropout Rate	{0.2, 0.3, 0.5, 0.7, 0.8}	0.5	Reduced overfitting while maintaining performance
Optimizer	{SGD, Adam, Nadam}	Adam	Achieved faster convergence and better performance
Activation Function	{ReLU, LeakyReLU, Tanh}	ReLU	Enabled efficient gradient flow
Epochs	{20, 50, 100, 150}	100	Sufficient for convergence

### Experimental results

This section narrates the findings of multiple proposed models in-depth and thoroughly analyzes how well they perform under different constraints. Three conceptual CNN schemes are proposed, deployed, and tested for effective malaria parasite classification: PCNN, SPCNN, and SFPCNN. A PCNN is a neural network architecture where multiple convolution layers operate concurrently, processing input data in parallel rather than sequentially. Since PCNN allows the processing of input data parallelly, it allows the extraction of more complex features at different levels of abstraction. A SPCNN is another type of neural network architecture where various parts or regions of images are processed parallelly by distinct branches of the network^46^. This architecture enables the network to focus on specific spatial regions of images independently, allowing it to extract the features of parasites effectively. Following the functional block in the Parallel Convolutional Network, a soft attention mechanism preferentially focuses on the most significant characteristics, improving the model’s ability to capture important spatial information. This attention mechanism dynamically weights the features, allowing the network to prioritize portions of the input that are critical to accurate categorization.

### Results of the PCNN model

**Fig. 8:**
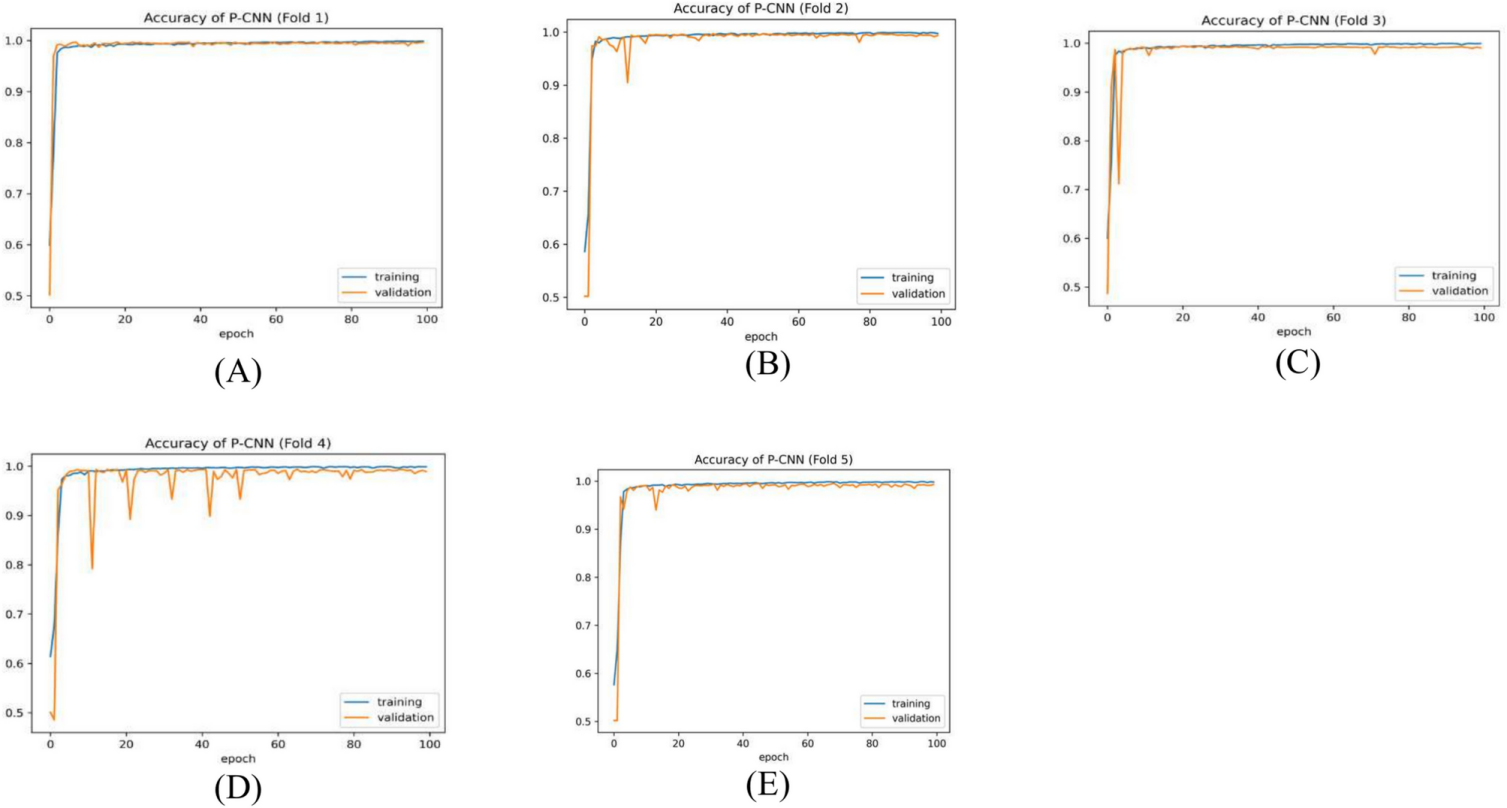
PCNN model curves of training and validation accuracy for malaria parasite classification.

**Fig. 9:**
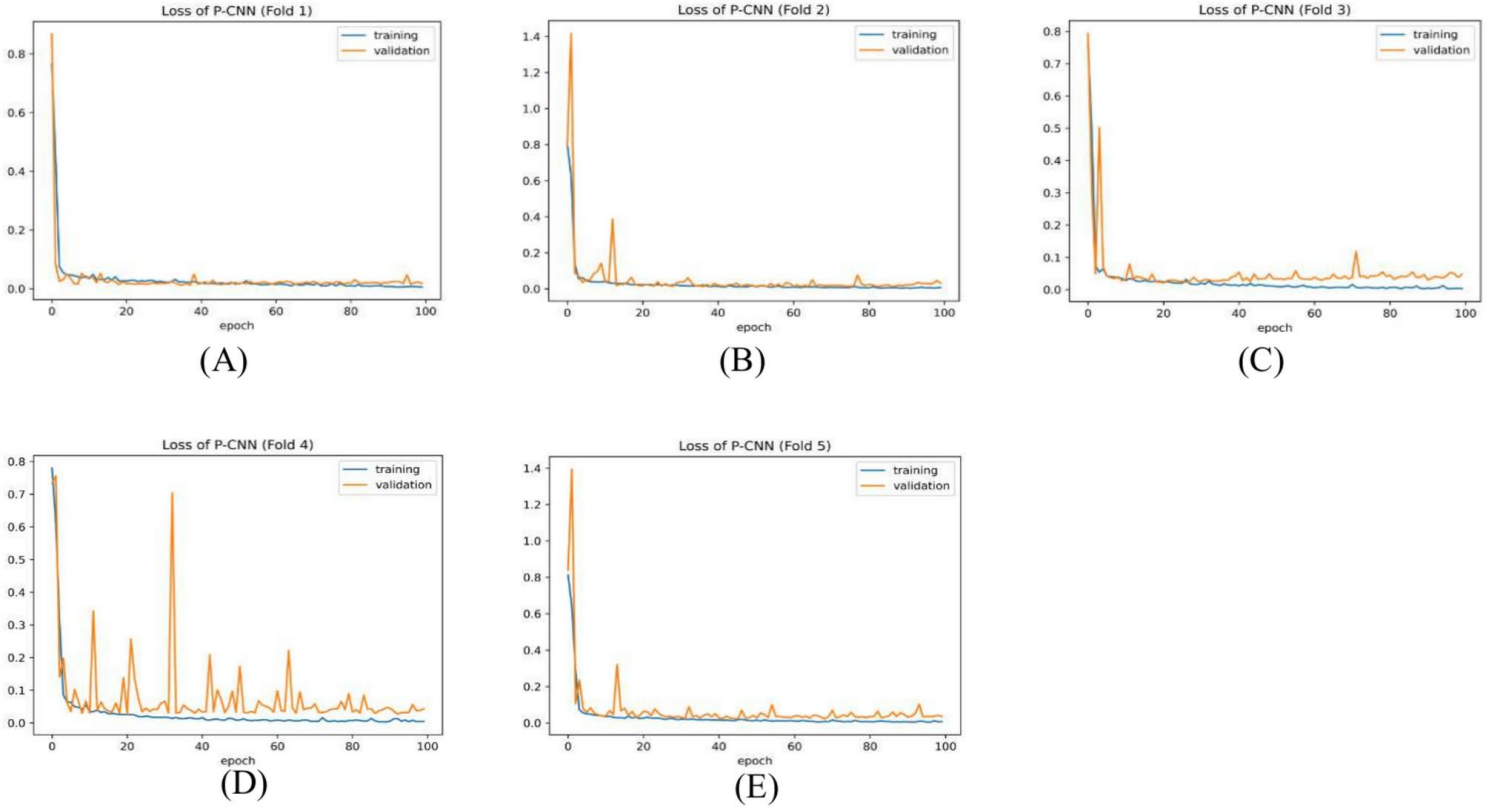
PCNN model loss curves for training and validation for malaria parasite classification.

**Fig. 10:**
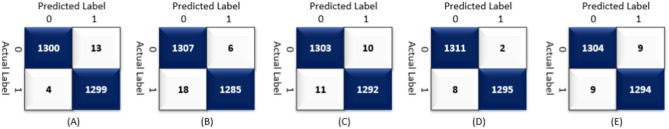
Confusion matrices (CMs) of the PCNN model during testing for malaria parasite classification.

**Fig. 11:**
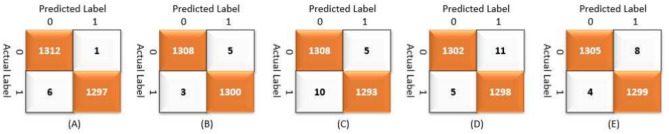
Confusion matrices (CMs) of the PCNN model during validation for malaria parasite classification.

**Fig. 12:**
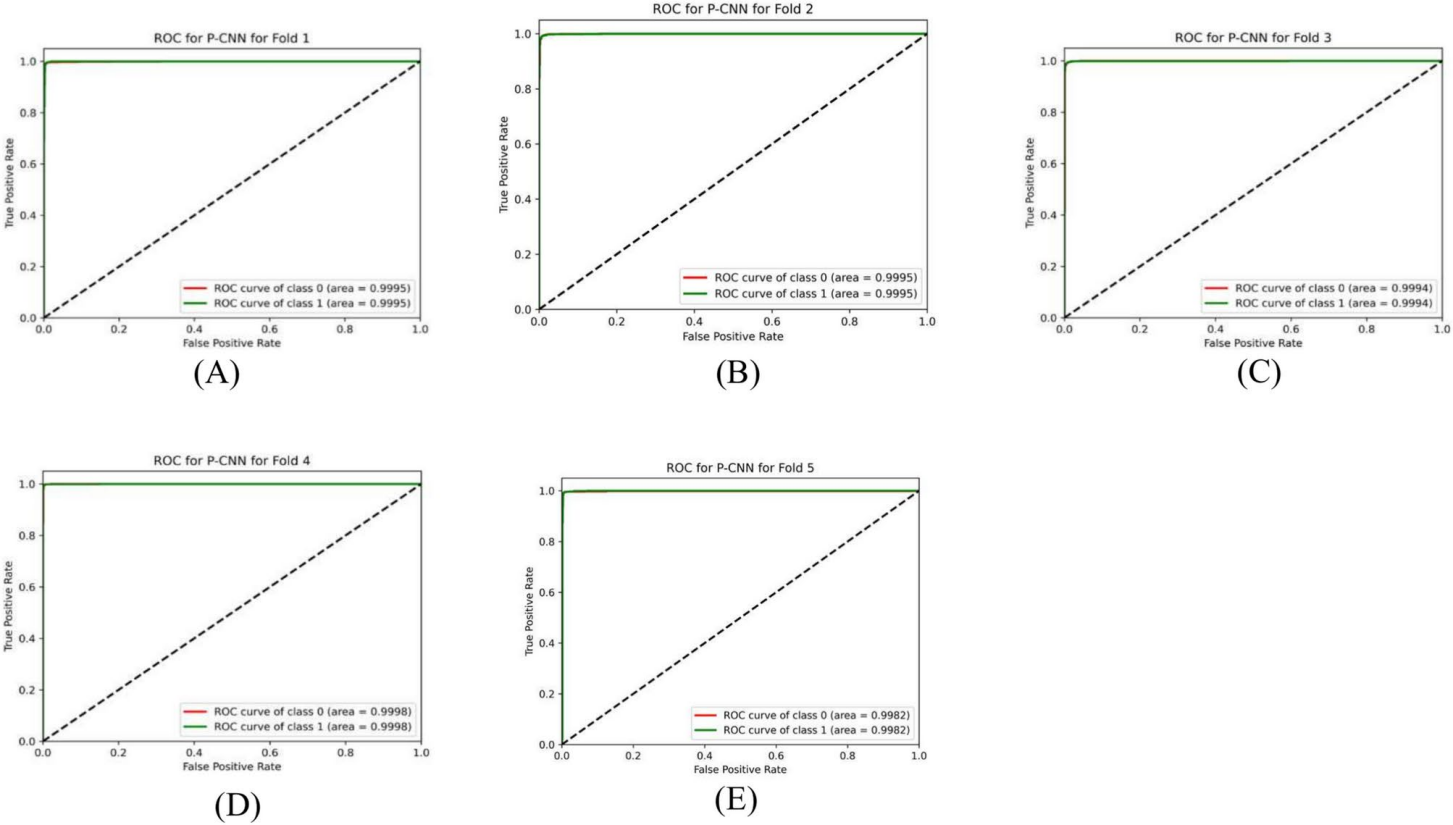
Class-wise ROC curves of the PCNN model malaria parasite classification.

Table 7 summarizes the PCNN’s class-wise performance on the test dataset. To guarantee the model’s accuracy was resilient and reliable, a rigorous five-fold cross-validation technique was used to verify its performance on varied subsets of data. Table 8 summarizes the class-wise performance of the PCNN on the validation set. The model demonstrated high precision, sensitivity, and f1-score of 99.31% ± 0.18, with a recognition rate of 99.31%. Figures 8 and 9 depict the accuracy and loss curves of the PCNN architecture. The network performed well throughout testing and validation, with accuracy rates of 99.31 ± 0.18 and 99.56 ± 0.13, respectively. These accuracy values show that the model can correctly categorize malaria parasites into their respective categories with great precision. The loss curve shows the fluctuation in losses throughout 100 epochs of training and validation. Figures 10 and 11 illustrate this approach by providing critical insights into categorization outcomes utilizing Confusion Matrices (CMs) for testing and validation, respectively. These matrices provide useful metrics such as precision, sensitivity, and the f1-score for each class. The performance of the PCNN model in distinguishing between classes is demonstrated by the ROC curve in Fig. 12. The AUC score of 99.92% ± 0.05 demonstrates the model’s exceptional ability to reliably categorize occurrences across various categories. This high AUC value indicates that the model efficiently balances the true positive vs false positive rate.

**Table 7:** Class-wise performance summary for PCNN with fivefold cross-validation on the test set.

Fold number	Classes	Precision (%)	Recall (%)	F1-score (%)	Support	Accuracy (%)	AUC (%)
Fold 1	Infected (0)	99.69	99.01	99.35	1313	99.35	99.94
	Uninfected (1)	99.01	99.69	99.35	1303		
	Macro Average	99.35	99.35	99.35	2616		
	Average	99.35	99.35	99.35	2616		
Fold 2	Infected (0)	98.64	99.54	99.09	1313	99.08	99.94
	Uninfected (1)	99.54	98.62	99.07	1303		
	Macro Average	99.09	99.08	99.08	2616		
	Average	99.09	99.08	99.08	2616		
Fold 3	Infected (0)	99.16	99.24	99.20	1313	99.20	99.93
	Uninfected (1)	99.23	99.16	99.19	1303		
	Macro Average	99.20	99.20	99.20	2616		
	Average	99.20	99.20	99.20	2616		
Fold 4	Infected (0)	99.39	99.85	99.62	1313	99.62	99.98
	Uninfected (1)	99.85	99.39	99.62	1303		
	Macro Average	99.62	99.62	99.62	2616		
	Average	**99.62**	**99.62**	**99.62**	2616		
Fold 5	Infected (0)	99.31	99.31	99.31	1313	99.31	99.82
	Uninfected (1)	99.31	99.31	99.31	1303		
	Macro Average	99.31	99.31	99.31	2616		
	Average	99.31	99.31	99.31	2616		
Average (µ) $\pm$ SD ($\sigma$) $( \%)$		99.31 $\pm$ 0.18	99.31 $\pm$ 0.18	99.31 $\pm$ 0.18	−	99.31 $\pm$ 0.18	99.92 $\pm$ 0.05

Significant values are in bold.

**Table 8:** Class-wise performance summary for PCNN with fivefold cross-validation on the validation set.

Fold Number	Classes	Precision (%)	Recall (%)	F1-score (%)	Support (%)	Accuracy (%)
Fold 1	Infected (0)	99.54	99.92	99.73	1313	99.73
	Uninfected (1)	99.92	99. 54	99.73	1303	
	Macro Average	99.73	99.73	99.73	2616	
	Average	99.73	99.73	99.73	2616	
Fold 2	Infected (0)	99.77	99.62	99.70	1313	99.69
	Uninfected (1)	99.62	99.77	99.69	1303	
	Macro Average	99.69	99.69	99.69	2616	
	Average	99.69	99.69	99.69	2616	
Fold 3	Infected (0)	99.24	99.62	99.43	1313	99.43
	Uninfected (1)	99.61	99.23	99.42	1303	
	Macro Average	99.43	99.43	99.43	2616	
	Average	99.43	99.43	99.43	2616	
Fold 4	Infected (0)	99.62	99.16	99.39	1313	99.39
	Uninfected (1)	99.16	99.62	99.39	1303	
	Macro Average	99.39	99.39	99.39	2616	
	Average	99.39	99.39	99.39	2616	
Fold 5	Infected (0)	99.69	99.39	99.54	1313	99.54
	Uninfected (1)	99.39	99.69	99.54	1303	
	Macro Average	99.54	99.54	99.54	2616	
	Average	99.54	99.54	99.54	2616	
Average (µ) $\pm$ SD ($\sigma$) $( \%)$		99.56 $\pm$ 0.13	99.56 $\pm$ 0.13	99.56 $\pm$ 0.13	−	99.56 $\pm$ 0.13

Results of the SPCNN model.

**Fig. 13:**
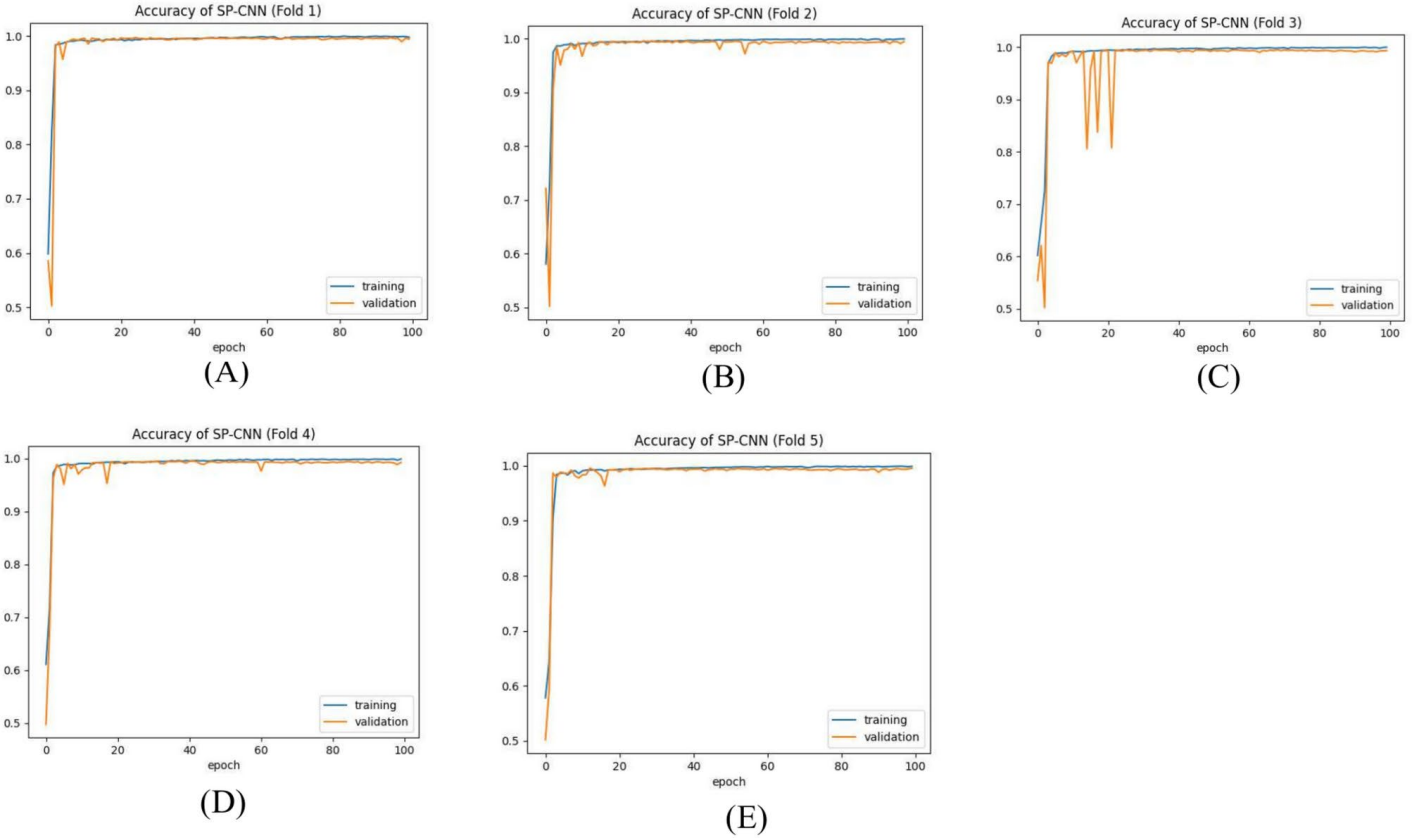
SPCNN model accuracy curves of training and validation for malaria parasite identification.

**Fig. 14:**
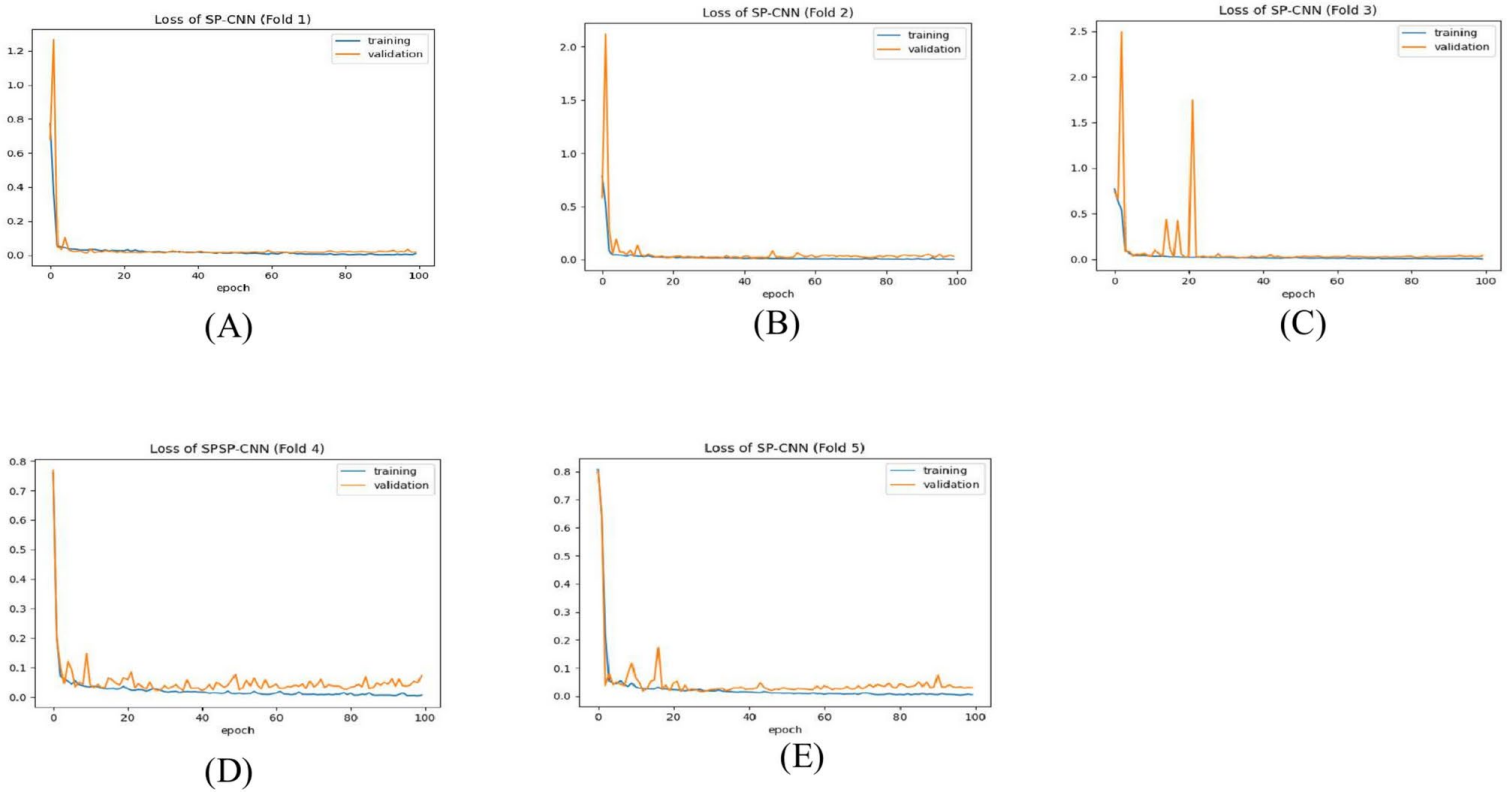
SPCNN model loss curves for training and validation for malaria parasite classification.

**Fig. 15:**
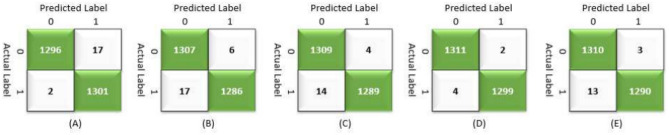
CMs of SPCNN model testing for malaria parasite classification.

**Fig. 16:**
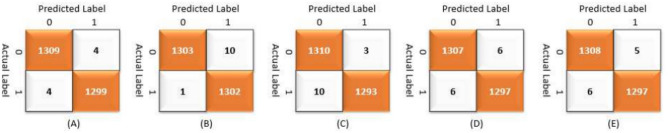
CMs of SPCNN model validation across folds (A) 1, (B) 2, (C) 3, (D) 4, and (E) 5 for malaria parasite classification.

**Fig. 17:**
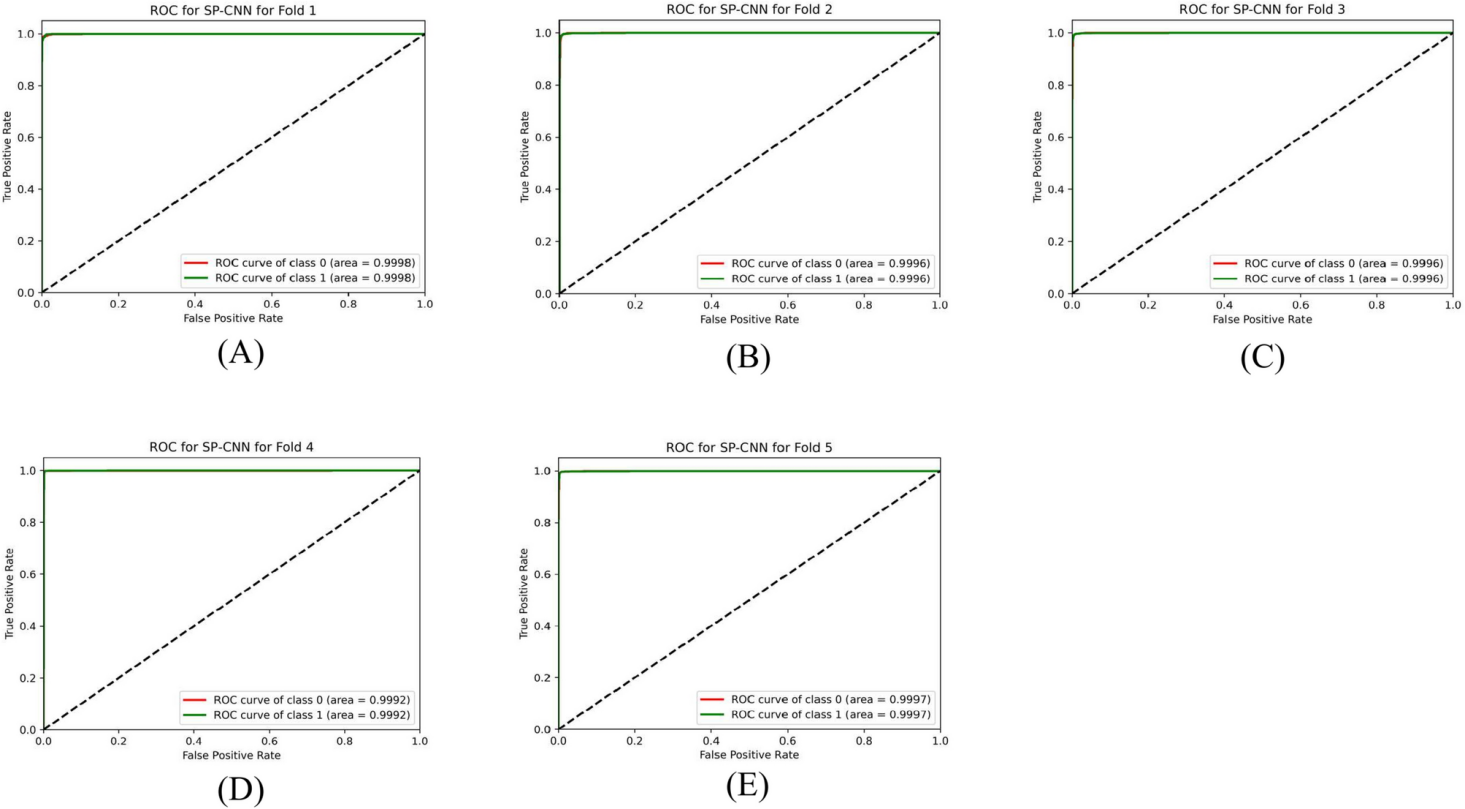
Class-wise ROCs of SPCNN across folds (A) 1, (B) 2, (C) 3, (D) 4, and (E) 5 for malaria parasite classification.

Table 9 presents the results of the SPCNN on the test set, while Table 10 shows its results on the validation set. A rigorous five-fold cross-checking method was applied to evaluate its effectiveness across several data subsets to ensure the model’s accuracy was dependable and robust. The model demonstrated high performance on both the validation and test sets, achieving a precision of 99.58% ± 0.06, recall and F1-score of 99.58% ± 0.06 on the validation set, and a precision of 99.38% ± 0.21, recall and F1-score of 99.37% ± 0.21 on the test set. The AUC was outstanding at 99.95 ± 0.01, with a margin of error of ± 0.05. Figures 13 and 14 show the accuracy and loss curves for the SPCNN. The network performed well in testing, with a classification accuracy of 99.37% ± 0.30 and a validation accuracy of 99.58% ± 0.06. The loss curve depicts the variations in loss over 100 epochs in both the training and validation stages. Figure 15 and 16 exhibit confusion matrices, while Tables 9 and 10 give extensive assessment metrics such as class-specific precision, recall, F1-score, and AUC. Figure 17 depicts the overall AUC-ROC values ranging from 0.9992 to 0.9997, efficiently discriminating between the infected (0) and uninfected (1) groups in binary classification.

**Table 9:** Class-wise Performance Summary for SPCNN on Test Set (Five folds CV).

Fold Number	Classes	Precision (%)	Recall (%)	F1-score (%)	Support	Accuracy (%)	AUC (%)
Fold 1	Infected (0)	99.85	98.71	99.27	1313	99.27	99.97
	Uninfected (1)	98.71	99.85	99.28	1303		
	Macro Average	99.28	99.28	99.27	2616		
	Average	99.28	99.28	99.27	2616		
Fold 2	Infected (0)	98.72	99.54	99.13	1313	99.12	99.95
	Uninfected (1)	99.54	98.70	99.11	1303		
	Macro Average	99.13	99.12	99.12	2616		
	Average	99.13	99.12	99.12	2616		
Fold 3	Infected (0)	98.94	99.70	99.32	1313	99.31	99.96
	Uninfected (1)	99.69	98.93	99.31	1303		
	Macro Average	99.32	99.31	99.31	2616		
	Average	99.32	99.31	99.31	2616		
Fold 4	Infected (0)	99.70	99.85	99.77	1313	99.77	99.92
	Uninfected (1)	99.85	99.69	99.77	1303		
	Macro Average	99.77	99.77	99.77	2616		
	Average	99.77	99.77	99.77	2616		
Fold 5	Infected (0)	99.02	99.77	99.39	1313	99.39	99.96
	Uninfected (1)	99.77	99.00	99.38	1303		
	Macro Average	99.39	99.39	99.39	2616		
	Average	99.39	99.39	99.39	2616		
Average (µ) $\pm$ SD ($\sigma$) $( \%)$		99.38 $\pm$ 0.21	99.37 $\pm$ 0.21	99.37 $\pm$ 0.21	−	99.37 $\pm$ 0.30	99.95 $\pm$ 0.01

**Table 10:** Class-wise Performance Summary for SPCNN on Validation Set (Five folds CV).

Fold Number	Classes	Precision (%)	Recall (%)	F1-score (%)	Support	Accuracy (%)
Fold 1	Infected (0)	99.70	99.70	99.70	1313	99.69
	Uninfected (1)	99.69	99.69	99.69	1303	
	Macro Average	99.69	99.69	99.69	2616	
	Average	99.69	99.69	99.69	2616	
Fold 2	Infected (0)	99.92	99.24	99.58	1313	99.58
	Uninfected (1)	99.24	99.92	99.58	1303	
	Macro Average	99.58	99.58	99.58	2616	
	Average	99.58	99.58	99.58	2616	
Fold 3	Infected (0)	99.24	99.77	99.51	1313	99.50
	Uninfected (1)	99.77	99.23	99.50	1303	
	Macro Average	99.51	99.50	99.50	2616	
	Average	99.51	99.50	99.50	2616	
Fold 4	Infected (0)	99.54	99.54	99.54	1313	99.54
	Uninfected (1)	99.54	99.54	99.54	1303	
	Macro Average	99.54	99.54	99.54	2616	
	Average	99.54	99.54	99.54	2616	
Fold 5	Infected (0)	99.54	99.62	99.58	1313	99.58
	Uninfected (1)	99.62	99.54	99.58	1303	
	Macro Average	99.58	99.58	99.58	2616	
	Average	99.58	99.58	99.58	2616	
Average (µ) $\pm$ SD ($\sigma$) $( \%)$		$99.58\pm$0.06	$99.58\pm$0.06	$99.58\pm$0.06	$-$	$99.58\pm$0.06

Results of SFPCNN model.

**Fig. 18:**
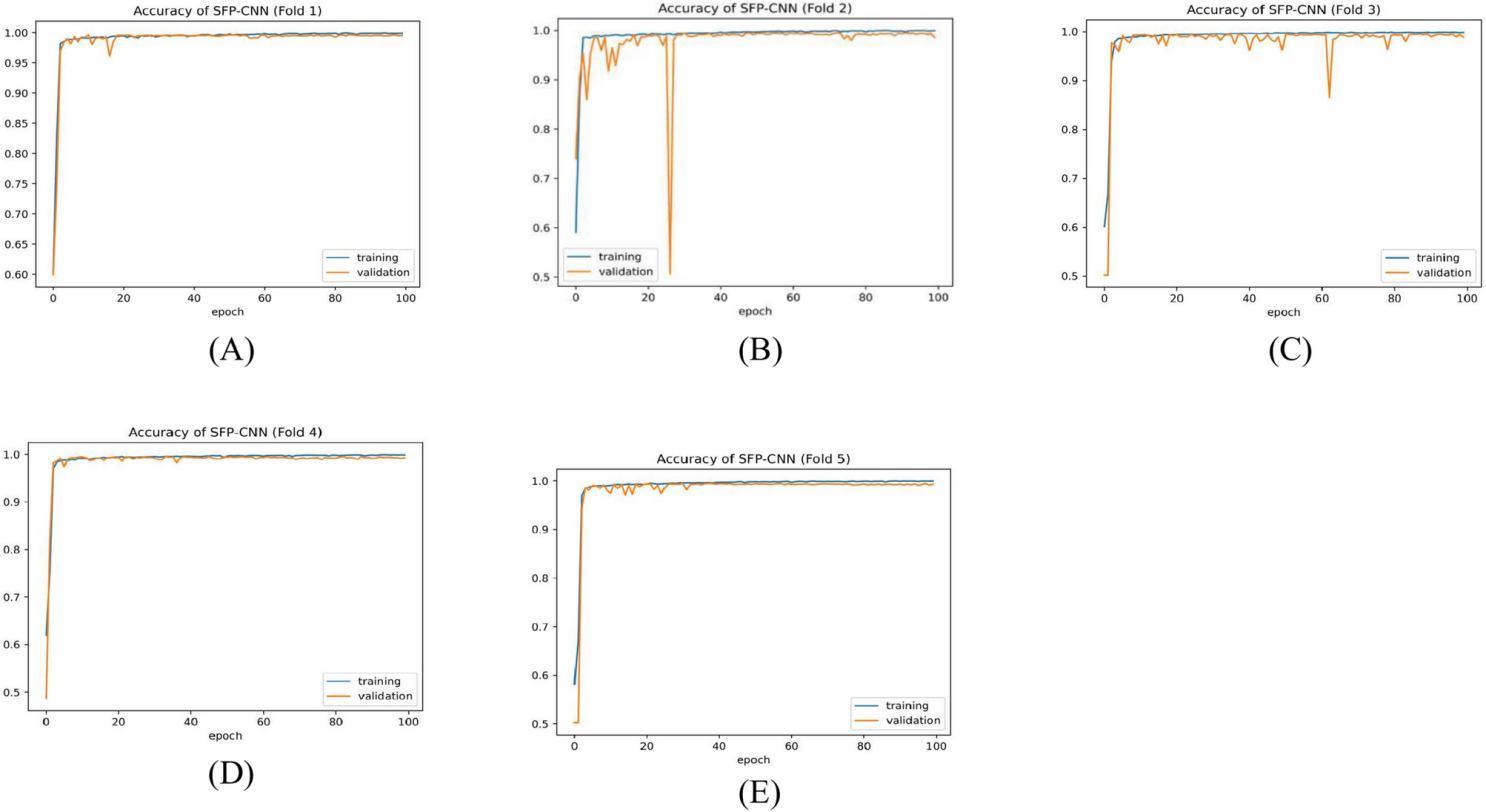
SFPCNN model accuracy curves for training and validation for malaria parasite classification.

**Fig. 19:**
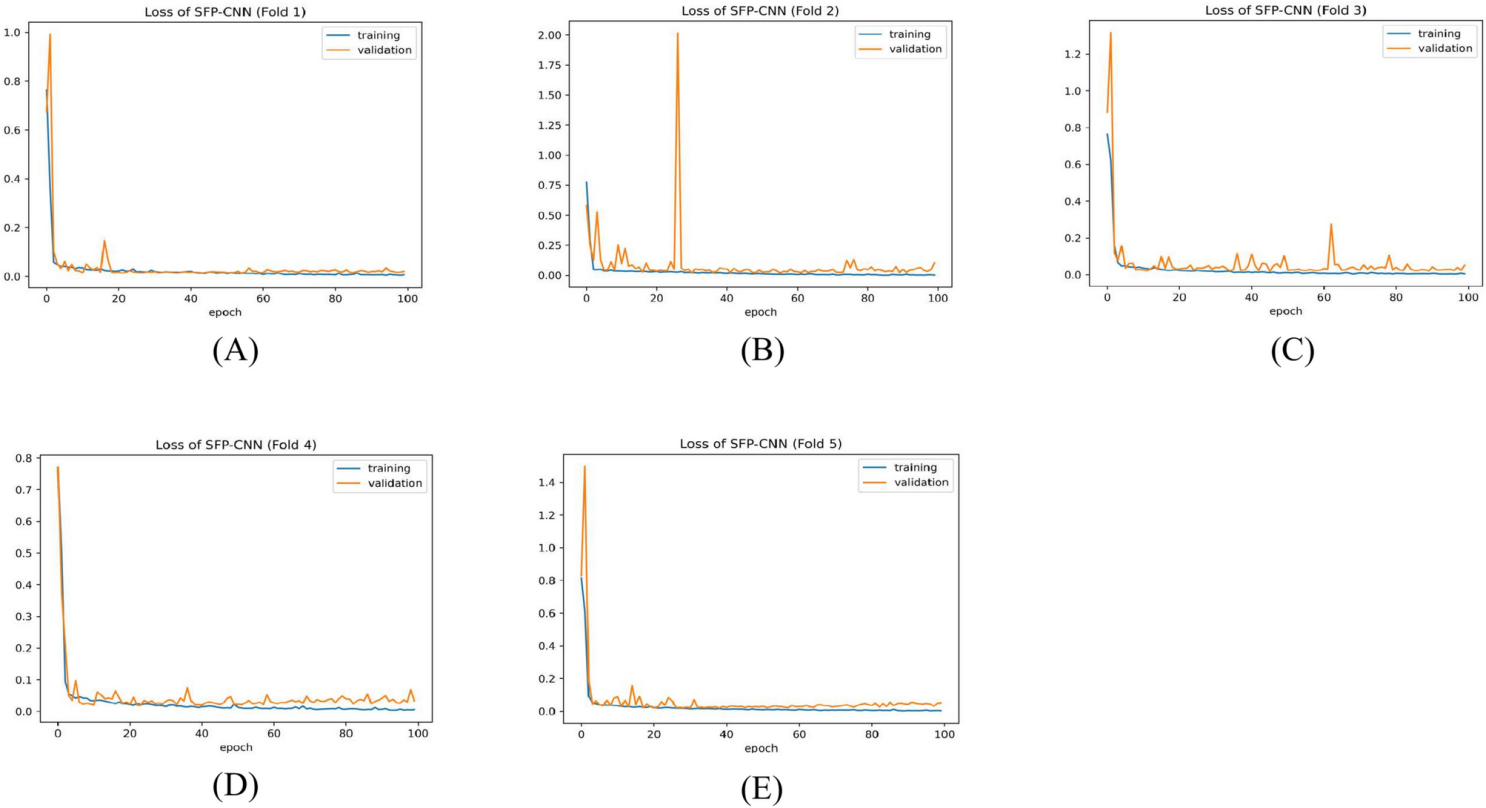
SFPCNN model loss curves for training and validation across folds (**A**) 1, (**B**) 2, (**C**) 3, (**D**) 4, and (E) 5 for malaria parasite classification.

**Fig. 20:**
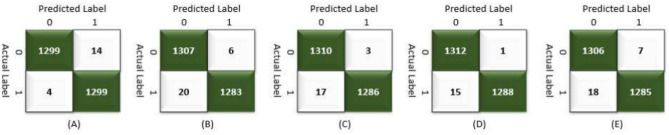
CMs of SFPCNN model testing across folds (**A**) 1, (**B**) 2, (**C**) 3, (**D**) 4, and (**E**) 5 for malaria parasite classification.

**Fig. 21:**
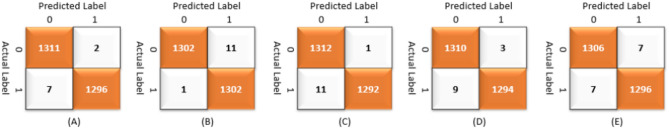
CMs of SFPCNN model validation across folds (**A**) 1, (**B**) 2, (**C**) 3, (**D**) 4, and (**E**) 5 for malaria parasite classification.

**Fig. 22:**
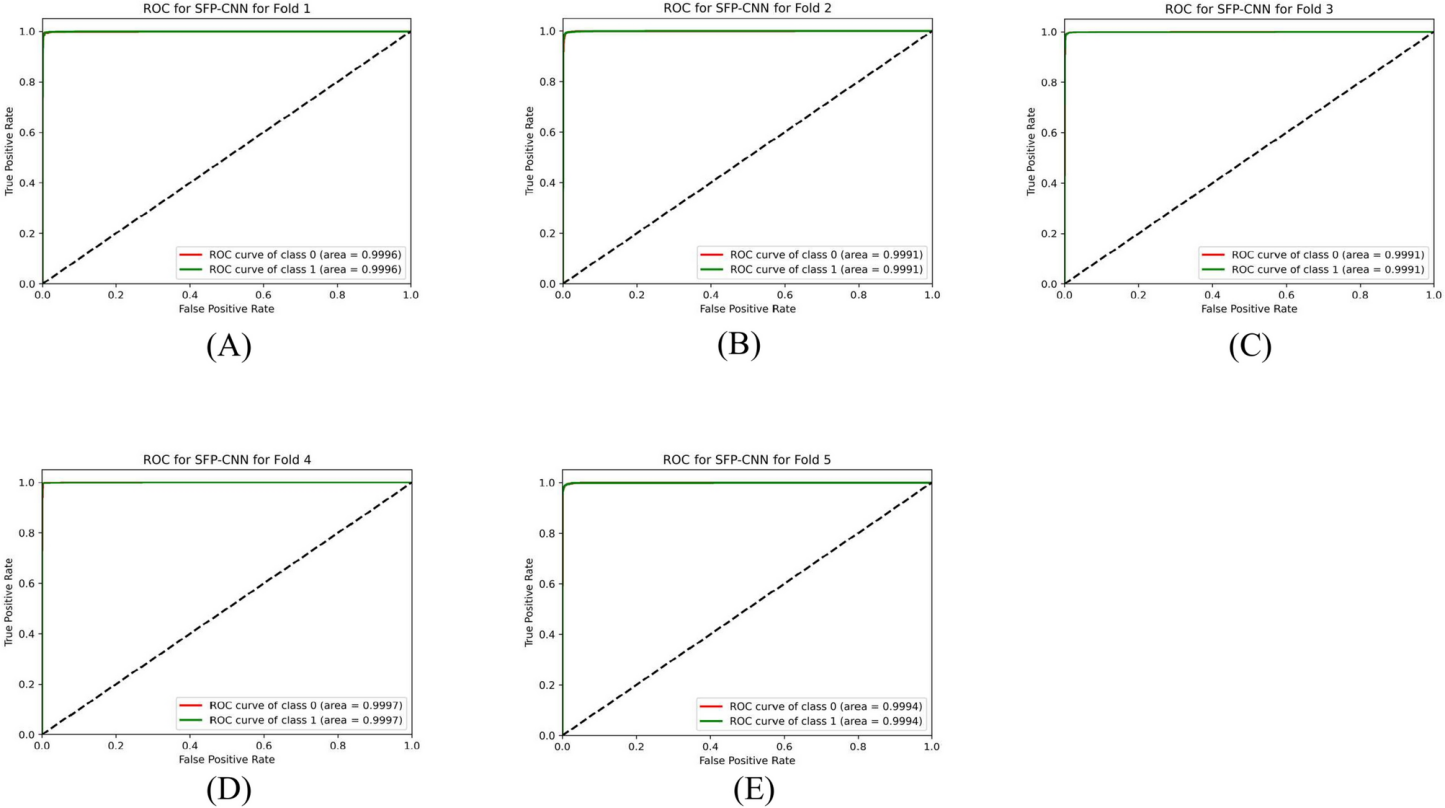
Class-wise ROCs of SFPCNN across folds (**A**) 1, (**B**) 2, (**C**) 3, (**D**) 4, and (**E**) 5 for malaria parasite classification.

This section outlines the results of malaria parasite classification using images of human red blood cells, distinguishing between parasitized and healthy states with the SFPCNN model. Table 11 summarizes the SFPCNN model’s performance across different classes on the test set, using five-fold cross-validation. The model consistently achieves excellent precision, sensitivity, and F1 metrics in parasitized and non-parasitized categories. The accuracy varies from 99.04% to 99.39%, with an average recognition rate of 99.19% ± 0.15. Table 12 shows the model’s performance across different classes on the validation set, which was also examined using five-fold cross-validation. The model maintains substantial precision, sensitivity, and F1-score across both categories, consistent with the test set. The average precision, sensitivity, and F1-score are 99.55% ± 0.06 in all folds. The success percentage ranges between 99.46% and 99.66%, with an average accuracy of 99.55% ± 0.06. Figures 18 and 19 display the model’s accuracy and loss during training and validation under five-fold cross-validation. Figures 20 and 21 show the confusion matrices and other performance metrics like class-wise accuracy, sensitivity, and F1-score for the testing and validation phases. The test set has an average precision of 99.20% ± 0.14, sensitivity of 99.19% ± 0.15, and F1 metric of 99.19% ± 0.15, whereas the validation set produces an average precision, sensitivity, and F1 metric of 99.55% ± 0.06. Figure 22 shows the AUC-ROC scores for every iteration of the five-part cross-validation. The AUC values range from 99.90% to 99.96%, with an average AUC of 99.93% ± 02.

**Table 11:** Class-wise Performance Summary for SFPCNN on Test Set (Five folds CV).

Fold number	Classes	Precision (%)	Recall (%)	F1-score (%)	Support	Accuracy (%)	AUC (%)
Fold 1	Infected (0)	99.69	98.93	99.31	1313	99.31	99.95
	Uninfected (1)	98.93	99.69	99.31	1303		
	Macro Average	99.31	99.31	99.31	2616		
	Average	99.31	99.31	99.31	2616		
Fold 2	Infected (0)	98.49	99.54	99.02	1313	99.01	99.90
	Uninfected (1)	99.53	99.47	99.00	1303		
	Macro Average	99.01	99.00	99.01	2616		
	Average	99.01	99.00	99.01	2616		
Fold 3	Infected (0)	98.72	99.77	99.24	1313	99.24	99.91
	Uninfected (1)	99.77	98.70	99.23	1303		
	Macro Average	99.24	99.23	99.24	2616		
	Average	99.24	99.23	99.24	2616		
Fold 4	Infected (0)	98.87	99.92	99.39	1313	99.39	99.96
	Uninfected (1)	99.92	98.85	99.38	1303		
	Macro Average	99.40	99.39	99.39	2616		
	Average	99.40	99.39	99.39	2616		
Fold 5	Infected (0)	98.64	99.47	99.05	1313	99.04	99.94
	Uninfected (1)	99.46	98.62	99.04	1303		
	Macro Average	99.05	99.04	99.04	2616		
	Average	99.05	99.04	99.04	2616		
Average (µ) $\pm$ SD ($\sigma$) $( \%)$		99.20 $\pm$ 0.14	99.19 $\pm$ 0.15	99.19 $\pm$ 0.15	-	99.19 $\pm$ 0.15	99.93 $\pm$ 0.02

**Table 12:** Class-wise Performance Summary for SFPCNN on Validation Set (Five folds CV).

Fold Number	Classes	Precision (%)	Recall (%)	F1-score (%)	Support	Accuracy (%)
Fold 1	Infected (0)	99.47	99.85	99.66	1313	99.66
	Uninfected (1)	99.85	99.46	99.65	1303	
	Macro Average	99.66	99.66	99.66	2616	
	Average	99.66	99.66	99.66	2616	
Fold 2	Infected (0)	99.92	99.16	99.54	1313	99.54
	Uninfected (1)	99.16	99.92	99.54	1303	
	Macro Average	99.54	99.54	99.54	2616	
	Average	99.54	99.54	99.54	2616	
Fold 3	Infected (0)	99.17	99.92	99.54	1313	99.54
	Uninfected (1)	99.92	99.16	99.54	1303	
	Macro Average	99.55	99.54	99.54	2616	
	Average	99.55	99.54	99.54	2616	
Fold 4	Infected (0)	99.32	99.77	99.54	1313	99.54
	Uninfected (1)	99.77	99.31	99.54	1303	
	Macro Average	99.54	99.54	99.54	2616	
	Average	99.54	99.54	99.54	2616	
Fold 5	Infected (0)	99.47	99.47	99.47	1313	99.46
	Uninfected (1)	99.46	99.46	99.46	1303	
	Macro Average	99.46	99.46	99.46	2616	
	Average	99.46	99.46	99.46	2616	
Average (µ) $\pm$ SD ($\sigma$) $( \%)$		99.55 $\pm$ 0.06	99.55 $\pm$ 0.06	99.55 $\pm$ 0.06	−	99.55 $\pm$ 0.06

### Performance comparison of malaria classification models

**Fig. 23:**
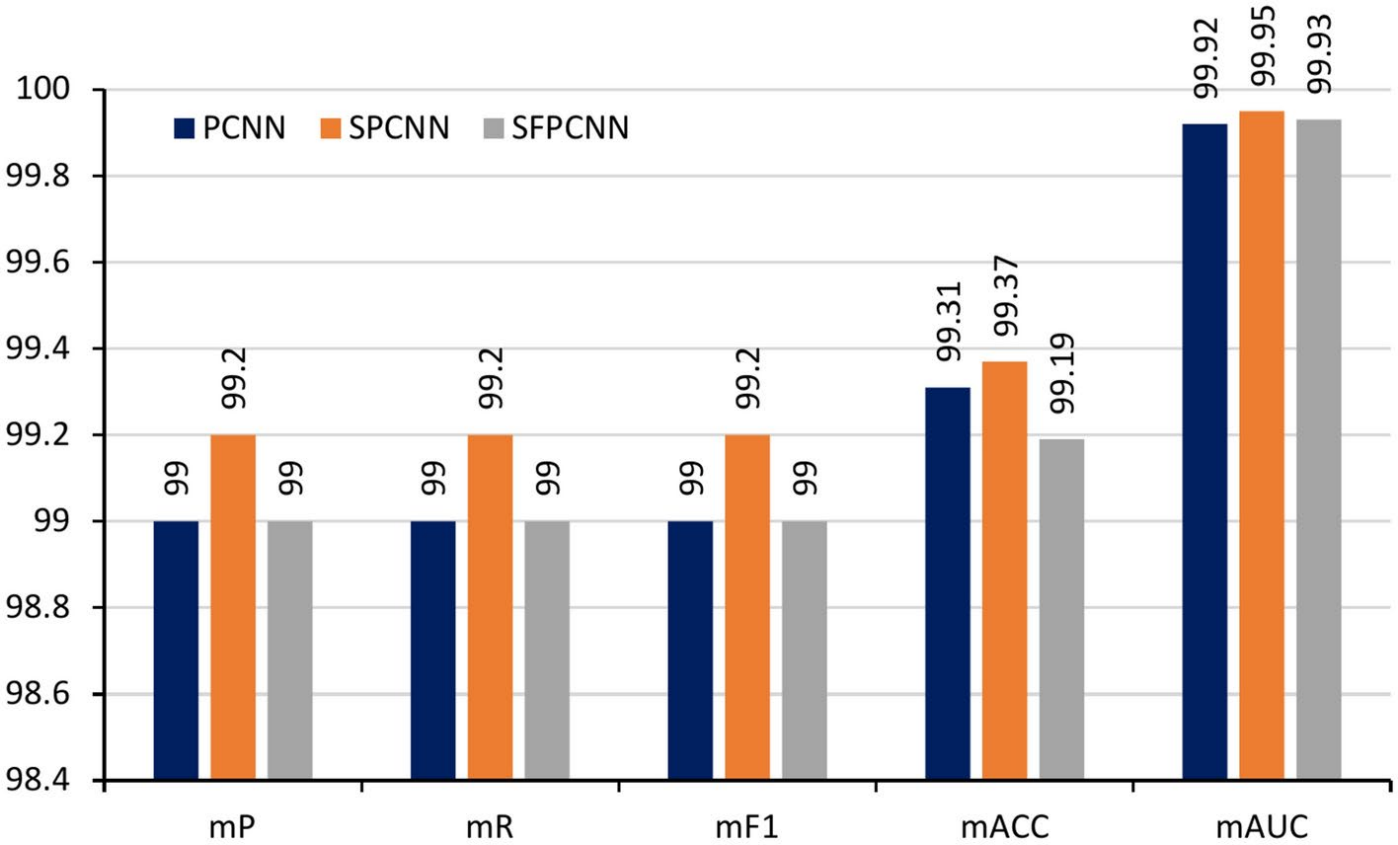
Comparison of performance on PCNN, SPCNN, and SFPCNN architecture.

The comparison of PCNN, SPCNN, and SFPCNN architectures shows that SPCNN consistently outperforms the other models in terms of accuracy and robustness. As shown in Fig. 23, SPCNN’s superior feature extraction capabilities lead to better classification results. The detailed metrics presented in Table 13 highlight that SPCNN achieves the highest mean precision (mP), mean recall (mR), mean F1 score (mF1), mean accuracy (mACC), and area under the curve (AUC), with bold values indicating the best-performing results. These findings demonstrate SPCNN’s effectiveness in malaria parasite detection.

**Table 13:** Performance Comparison of Malaria Classification Models Using Cross-Validation (mP = mean precision, mR = mean Recall, mF1 = mean f1 score, mACC = mean accuracy, AUC = area under the curve).

CV		mP (%)	mR (%)	mF1 (%)	mACC (%)	AUC (%)
Fold 1	PCNN	**99.35**	**99.35**	**99.35**	**99.35**	99.94
	SPCNN	99.28	99.28	99.27	99.27	**99.97**
	SFPCNN	99.31	99.31	99.31	99.31	99.95
Fold 2	PCNN	99.09	99.08	99.08	99.08	99.94
	SPCNN	**99.13**	**99.12**	**99.12**	**99.12**	**99.95**
	SFPCNN	99.01	99.00	99.01	99.01	99.90
Fold 3	PCNN	99.20	99.20	99.20	99.20	99.93
	SPCNN	**99.32**	**99.31**	**99.31**	**99.31**	**99.96**
	SFPCNN	99.24	99.23	99.24	99.24	99.91
Fold 4	PCNN	99.62	99.62	99.62	99.62	99.98
	SPCNN	**99.77**	**99.77**	**99.77**	**99.77**	99.92
	SFPCNN	99.40	99.39	99.39	99.39	99.96
Fold 5	PCNN	99.31	99.31	99.31	99.31	99.82
	SPCNN	**99.39**	**99.39**	**99.39**	**99.38**	**99.96**
	SFPCNN	99.05	99.04	99.04	99.04	99.94

*Bold values indicate best results.

### Benchmarking the SPCNN approach against TL models

This section evaluates the proposed SPCNN method’s performance in malaria parasite classification by comparing it with SOTA TL models, including VGG16, ResNet152, MobileNetV3Small, EfficientNetB6, EfficientNetB7, DenseNet201, ViT, DeiT, ImageIntern, and Swin Transformer variants (v1 and v2). Five key metrics, Precision, Recall, F1 Score, Accuracy, and AUC, were used for this benchmarking.

The SPCNN method achieved best results, scoring Precision (99.38%), Recall (99.37%), and F1 Score (99.37%), reflecting its ability to correctly classify malaria parasites with no false positives or false negatives. The Accuracy of SPCNN was measured at 99.37%, with an AUC of 99.95%, surpassing all other models by a significant margin. It outperformed all standard TL models, with VGG16 and DenseNet201 emerging as the closest competitors, achieving a performance score (Precision, Recall, and F1 Score) around 97.55%. Among the transformer-based models, ViT, ImageIntern, and Swin Transformer v2 achieved relatively high performance, with Precision, Recall, and F1 Scores in the range of 0.97%–0.98% and Accuracy greater than 98%. However, the SPCNN model still outperformed these models by maintaining higher performance metrics.

This comprehensive benchmarking highlights the superiority of SPCNN in identifying malaria parasites, showcasing its robustness and accuracy over widely used TF models. The results emphasize the effectiveness of SPCNN in achieving the best possible outcomes across all performance metrics, as summarized in Table 14**.**

**Table 14:** Performance comparison of the proposed model with various TL models (*Bold values indicate best results).

Model	Precision (%)	Recall (%)	F1 Score (%)	Accuracy (%)	AUC (%)
VGG16	97.54	97.56	97.55	97.55	99.39
ResNet152	87.68	87.70	87.69	87.69	94.37
MobileNetV3Small	63.10	63.12	63.11	63.12	66.54
EfficientNetB6	59.21	59.24	59.22	59.22	61.94
EfficientNetB7	61.15	61.10	61.13	61.13	64.82
DenseNet201	97.55	97.55	97.55	97.55	99.23
ViT	98.27	98.26	98.27	98.28	99.11
DeiT	98.75	98.73	98.74	98.74	99.38
ImageIntern	98.23	98.22	98.23	98.24	99.05
Swin Transformer v1	97.74	97.73	97.73	97.74	98.37
Swin Transformer v2	98.06	98.05	98.05	98.05	99.42
**Proposed (SPCNN)**	**99.38**	**99.37**	**99.37**	**99.37**	**99.95**

### Comparison of computational recourses

Three CNN architectures are compared in detail in Table 15**,** with performance across multiple criteria being highlighted: PCNN, SPCNN, and SFPCNN. With 3.518 million parameters and a 42-megabyte model size, SFPCNN is the most complex model, followed by SPCNN with 2.207 million and PCNN with 1.878 million. Despite its added complexity, SFPCNN is not the most efficient model; SPCNN is. It also outperforms the other two models’ inference speed, with an average testing time of just 0.00252 s per instance. Despite using the smallest model size (22 megabytes) and having the fewest parameters, PCNN requires more testing and training than SPCNN. The number of convolutional layers in all three models is eight, suggesting a similar network depth; however, the architecture of SPCNN is tuned for greater computing efficiency. Although the complexity of SFPCNN may indicate possible benefits, the performance gains are negligible, making it less advantageous than SPCNN. As a result, SPCNN is shown to be the best model out of the three. It achieves the best balance between computational efficiency and model complexity, making it the best option for applications where accuracy and speed are equally important.

**Table 15:** Computational resource comparison among evaluated methods).

Performance Criteria	PCNN	SPCNN	SFPCNN
Total Parameters (Million)	**1.878**	2.207	3.518
Trainable Parameters (Million)	**1.876**	2.205	3.516
Number of Layers (CL)	8	8	8
Size (Megabytes)	**22**	26	42
Average Testing Time (seconds)	0.00263	0.00252	0.00266

*Bold values indicates best results

### Performance comparison of the proposed model with other recent SOTA methods

The proposed SPCNN model for malaria parasite classification exceeds existing state-of-the-art algorithms, as shown in Table 16. The SPCNN model outperforms existing approaches by achieving almost perfect scores in precision (99.20%), sensitivity (99.20%), and F1 score (99.20%), as well as 99.37% accuracy and 99.95% AUC. For instance, while Yang et al.'s Customized CNN and Islam et al.'s Transformer model are robust, they must catch up to the proposed SPCNN regarding precision and sensitivity. Similarly, while Alqudah et al.'s CNN and G. Madhu et al.'s Inception V3 perform brilliantly, they do not equal the SPCNN’s flawless Classification criteria. Models such as Montalbo et al.'s EfficientNetB0 and Efaz et al.'s CNN-VGG19-ResNet50 produce impressive results, but they fall short of the SPCNN in precision and overall accuracy. Even Rajaraman et al.'s VGG19-SqueezeNet-InceptionResNetV2, which has great precision, is surpassed by the SPCNN’s superior parameters. Zhu et al.'s ROENet, another high-performing model, is outperformed by the SPCNN in all metrics. This comprehensive comparison demonstrates the SPCNN model’s remarkable accuracy and effectiveness, defining a new standard for parasitic malaria categorization.

**Table 16:** Performance comparison of the proposed model with previously developed SOTA methods

References	Methods	Optimizer	Learning rate	Precision (%)	Recall (%)	F1 score (%)	Accuracy (%)	AUC (%)
Yang et al.^47^	Customized CNN	-	0.001	94.25	92.59	-	93.46	98.39
Islam et al.^48^	Transformer	SGD	0.001	96.99	95.88	-	96.41	99.11
Alqudah et al.^49^	CNN	Adam	0.001	-	98.79	-	98.85	-
G. Madhu et al.^2^	Inception V3	Adam	-	-	**99.57**	**99.36**	99.35	99.73
Montalbo et al.^50^	EfficientNetB0	Adam	0.0001	93.87	95.46	94.66	94.70	-
Efaz et al.^51^	CNN-VGG19-Resnet50	-	0.0005	-	96.0	96.7	96.7	-
Rajaraman et al.^52^	VGG19-SqueezeNet-InceptionResNetV2	SGD	0.0001	**99.67**	-	99.1	99.11	98.94
Zhu et al ^53^	ROENet	SGD	0.0001	-	94.79	95.69	95.73	-
**Proposed**	**SPCNN**	**Adam**	**0.0001**	**99.38**	**99.37**	**99.37**	**99.37**	**99.95**

*Bold values indicate best results.

### Multi-species and stage-wise classification

Multistage classification evaluates the performance of malaria detection models across species and stages of malaria parasites using the MP-IDB dataset^54^ which provides a diverse representation of malaria parasite species and their developmental stages, enabling a comprehensive evaluation of classification models. This approach allows for a comprehensive analysis of the model’s effectiveness in identifying key variations in different malaria categories, thereby ensuring accurate diagnostic predictions.

### Species wise performances

Table 17 presents detailed performance metrics, including precision, recall, F1-score, accuracy, and AUC, for PCNN, SFPCNN, and SPCNN. These models were evaluated based on their ability to classify four species of malaria parasites: Falciparum, Malariae, Ovale, and Vivax.

**Table 17:** Species-wise performance comparisons among the models.

Implemented Methods	Species	Precision (%)	Recall (%)	F1-score (%)	Accuracy (%)	AUC (%)
PCNN	Falciparum (Class 3)	98.47	97.77	98.12	94.67	96.44
	Malariae (Class 1)	93.55	94.83	94.19		
	Ovale (Class 0)	90.74	90.13	90.43		
	Vivax (Class 2)	97.32	97.45	97.38		
SFPCNN	Falciparum (Class 3)	98.44	97.47	97.95	93.94	95.56
	Malariae (Class 1)	92.53	92.16	92.34		
	Ovale (Class 0)	87.86	87.42	87.64		
	Vivax (Class 2)	96.07	96.59	96.33		
SPCNN	Falciparum (Class 3)	99.46	99.34	99.40	95.78	97.59
	Malariae (Class 1)	95.22	94.68	94.95		
	Ovale (Class 0)	90.83	90.39	90.61		
	Vivax (Class 2)	98.15	97.61	97.88		

Among the models, the SPCNN (proposed model) consistently outperformed PCNN and SFPCNN across all metrics. For Falciparum (Class 3), the model achieved a precision of 99.46%, a recall of 99.34%, and an F1-score of 99.40%. Similarly, Malariae (Class 1) attained an F1-score of 94.95%. Despite being a challenging class, Ovale (Class 0) demonstrated substantial improvement in detection, with the proposed model achieving an F1-score of 90.61%. The model provided a precise classification for Vivax (Class 2), achieving an F1-score of 97.88%. Additionally, the SPCNN achieved an overall AUC of 97.59% and an accuracy of 95.78%.

**Fig. 24:**
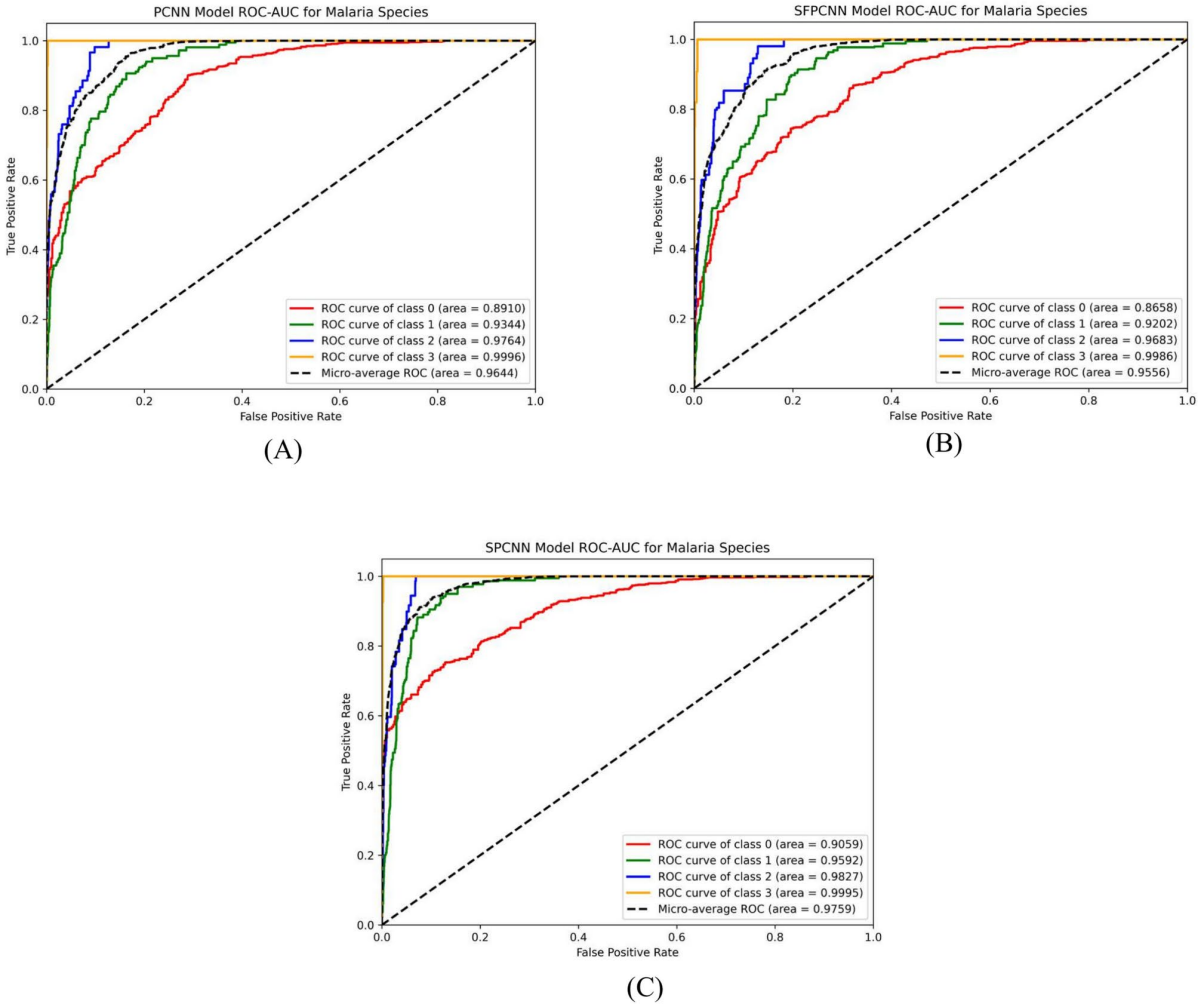
Class-wise ROCs for Falciparum, Malariae, Ovale, and Vivax across (**A**) PCNN, (**B**) SFPCNN, and (C) SPCNN for malaria parasite Species classification.

Figure 24 illustrates the species-wise ROC curves for each model. The ROC-AUC scores indicate that the SPCNN model (Fig. 24C) is generally more reliable. For Falciparum, the model achieved an AUC of 97.59%, while Vivax achieved an AUC of 93.63%. These higher AUC values for Falciparum and Vivax are attributed to their larger sample sizes, which provide the model with sufficient data to learn distinguishing features effectively. These results highlight the SPCNN’s robustness and precision in species-specific malaria classification, outperforming PCNN and SFPCNN across most metrics.

### Stage wise performances

The classification was further extended to assess the stages of malaria parasites: Ring, Trophozoite, Schizont, and Gametocyte for each species. Table 18 outlines the models’ stage-wise performance, demonstrating their nuanced ability to identify the parasites’ developmental stages.

**Table 18:** Stage-wise performance comparisons among the models.

Implemented methods	Species	Stages	Precision (%)	Recall (%)	F1-score (%)	Accuracy (%)	AUC (%)
PCNN	Falciparum	Ring (Class 2)	94.81	93.03	93.91	91.07	92.13
		Trophozoite (Class 1)	92.15	92.11	92.13		
		Schizont (Class 0)	84.43	83.65	84.04		
	Malariae	Trophozoite (Class 2)	95.32	94.89	95.10	89.85	91.74
		Schizont (Class 1)	90.11	89.27	89.69		
		Gametocyte (Class 0)	85.69	84.40	85.04		
	Ovale	Ring (Class 0)	85.92	85.18	85.55	90.18	91.29
		Trophozoite (Class 2)	94.66	94.32	94.49		
		Gametocyte (Class 1)	89.74	89.73	89.74		
	Vivax	Ring (Class 3)	97.55	97.01	97.28	91.73	92.55
		Trophozoite (Class 1)	86.73	86.42	86.57		
		Schizont (Class 2)	95.08	94.59	94.83		
		Gametocyte (Class 0)	82.64	82.02	82.33		
SFPCNN	Falciparum	Ring (Class 2)	94.13	93.41	93.77	90.46	92.07
		Trophozoite (Class 1)	91.71	91.39	91.55		
		Schizont (Class 0)	84.01	82.22	83.11		
	Malariae	Trophozoite (Class 2)	95.19	94.62	94.90	90.17	91.41
		Schizont (Class 1)	89.33	89.18	89.25		
		Gametocyte (Class 0)	85.49	85.29	85.39		
	Ovale	Ring (Class 0)	85.80	85.03	85.41	90.23	91.52
		Trophozoite (Class 2)	94.03	93.47	93.75		
		Gametocyte (Class 1)	89.15	89.55	89.35		
	Vivax	Ring (Class 3)	96.44	96.39	96.41	91.32	92.23
		Trophozoite (Class 1)	85.61	84.56	85.08		
		Schizont (Class 2)	94.42	94.30	94.36		
		Gametocyte (Class 0)	82.19	82.00	82.09		
SPCNN	Falciparum	Ring (Class 2)	95.73	96.24	95.98	91.75	93.06
		Trophozoite (Class 1)	92.61	92.98	92.79		
		Schizont (Class 0)	85.03	85.72	85.37		
	Malariae	Trophozoite (Class 2)	96.01	96.06	96.03	91.34	92.55
		Schizont (Class 1)	90.46	90.06	90.26		
		Gametocyte (Class 0)	86.13	85.23	85.68		
	Ovale	Ring (Class 0)	86.38	86.94	86.66	90.55	92.73
		Trophozoite (Class 2)	95.68	95.80	95.74		
		Gametocyte (Class 1)	90.76	90.90	90.83		
	Vivax	Ring (Class 3)	98.42	98.31	98.36	91.87	93.63
		Trophozoite (Class 1)	87.87	87.62	87.74		
		Schizont (Class 2)	95.91	95.55	95.73		
		Gametocyte (Class 0)	83.35	83.44	83.39		

The SPCNN excelled in detecting all stages, particularly the Ring stage of Falciparum (Class 2), achieving a precision of 95.73, a recall of 96.24, and an F1-score of 95.98. For the Trophozoite stage (Class 1), the model achieved a notable F1-score of 92.79. Similarly, the Schizont stage (Class 0) was effectively classified, attaining an F1-score of 85.37. For Vivax, the model demonstrated exemplary performance in identifying the Ring stage, with a precision of 98.42, a recall of 98.31, and an F1-score of 98.36. The Schizont stage of Vivax (Class 2) also showed consistent results, with the SPCNN outperforming other models in all metrics.

**Fig. 25:**
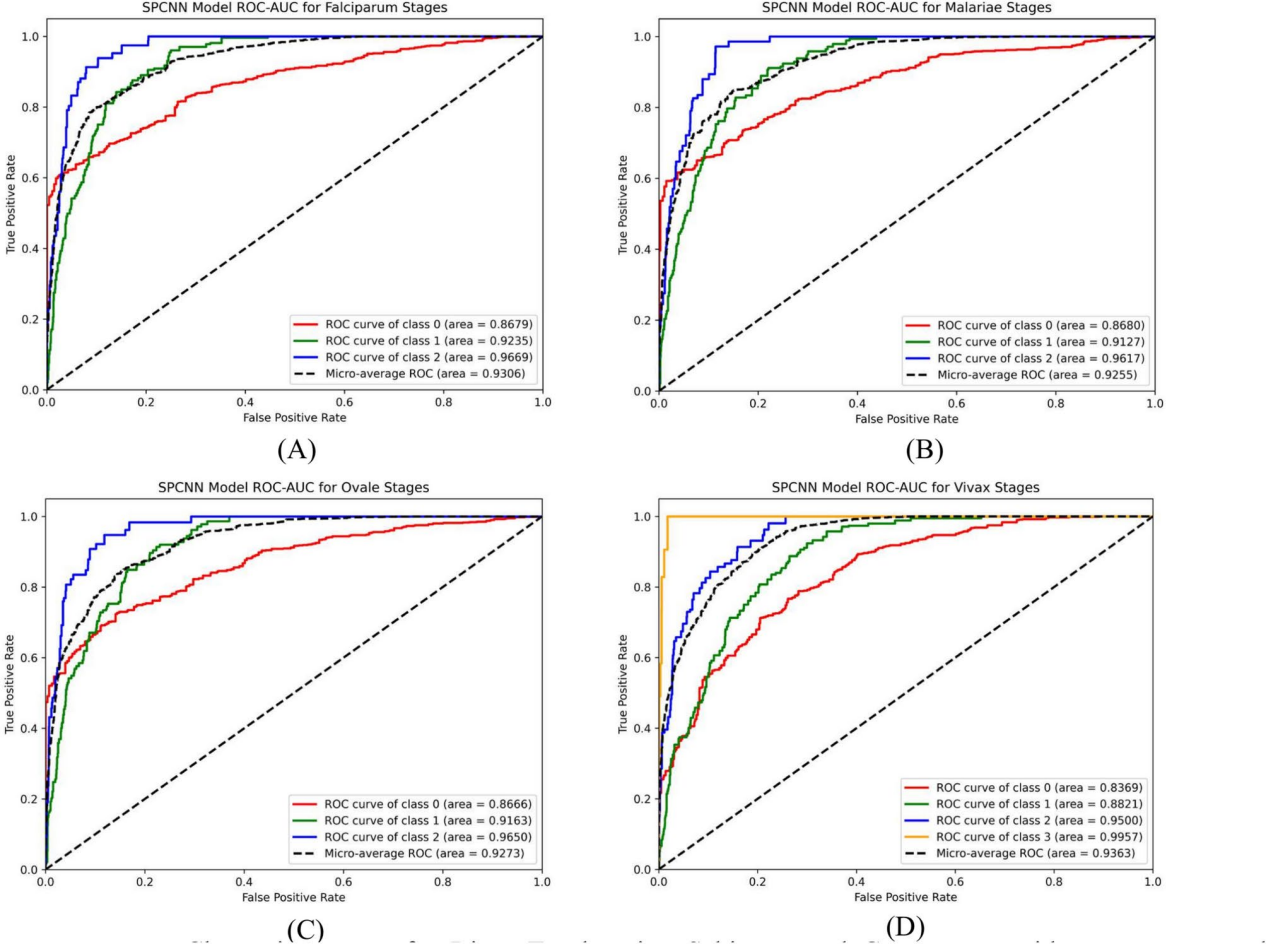
Class-wise ROCs for Ring, Trophozoite, Schizont, and Gametocyte with SPCNN on malaria parasite’s different stages classification.

The analysis highlights the critical role of sample size in influencing performance scores. Larger sample sizes enable more reliable and consistent performance across various stages. These findings are visually supported by the stage-specific ROC curves (Fig. 25), which confirm the proposed model’s superiority in capturing stage-specific characteristics. This ability to accurately classify species and stages reinforces the SPCNN’s applicability as a diagnostic tool for malaria parasite identification.

### External validation

**Fig. 26:**
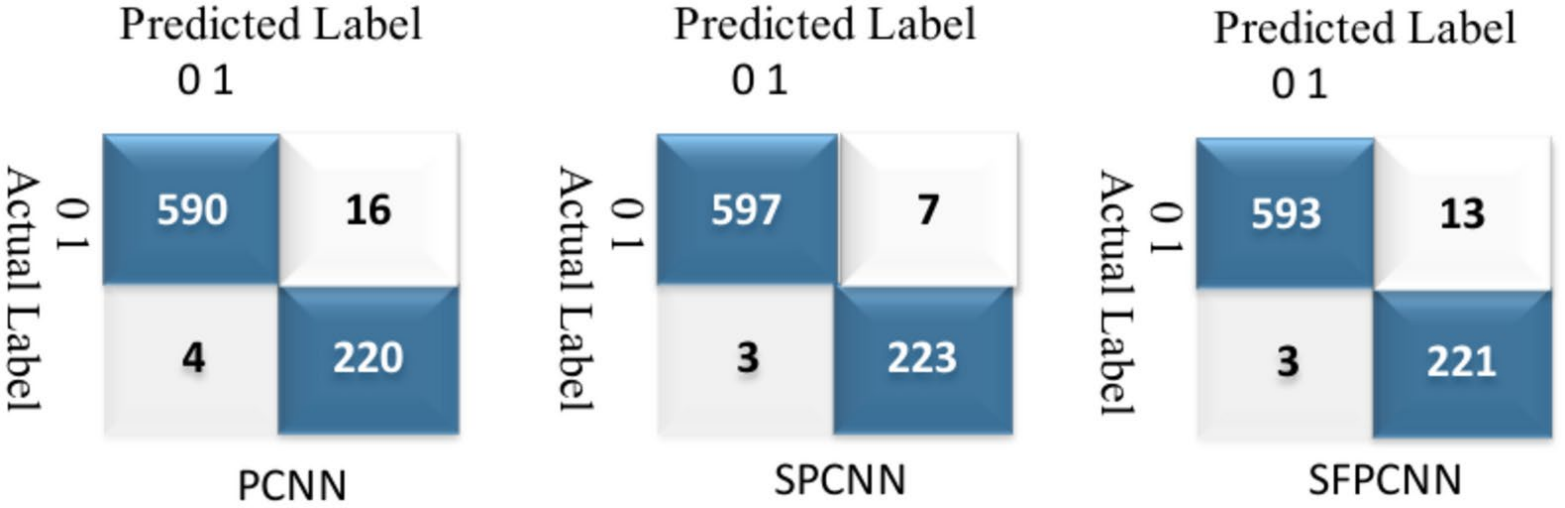
CMs of the three implemented methods on an external dataset of Plasmodium falciparum-infected (0) vs uninfected (1) samples.

**Fig. 27:**
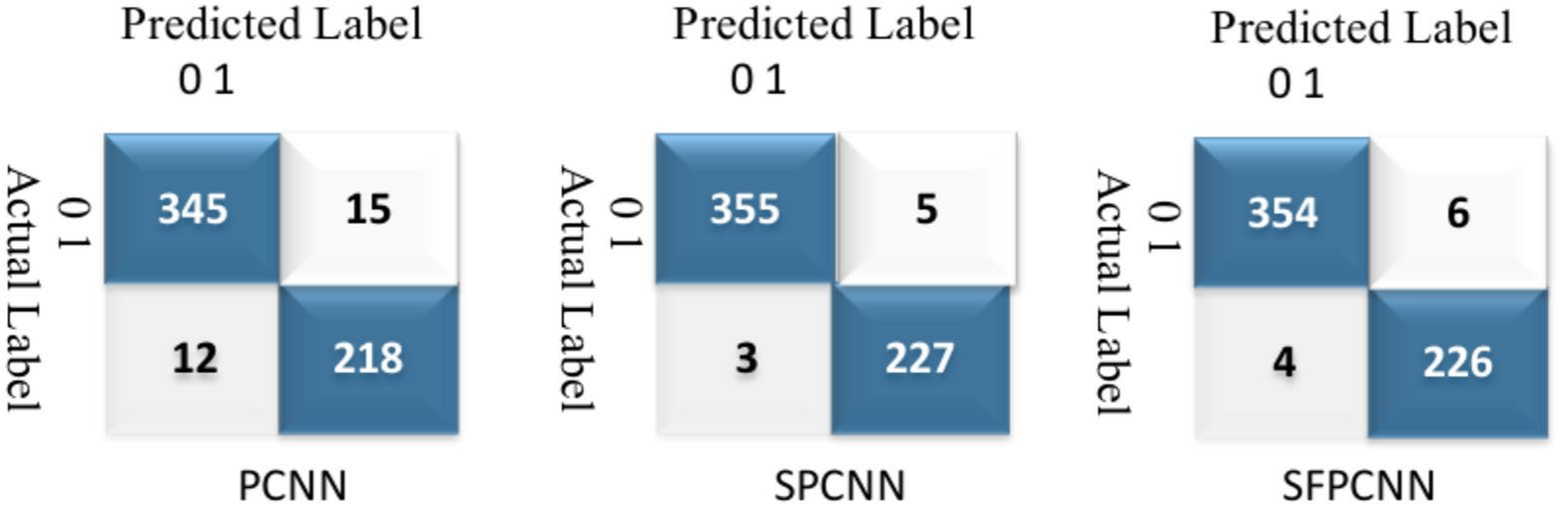
CMs of the three implemented methods on an external dataset of Plasmodium vivax-infected (0) vs uninfected (1) samples.

In the performance evaluation of two external datasets, Plasmodium falciparum-infected^55^ and Plasmodium vivax-infected^56^ SPCNN was identified as the most efficient method. For the three models, each comprising 150 infected and 50 healthy patients, Figs. 26 and 27 illustrate confusion matrices.

**Fig. 28:**
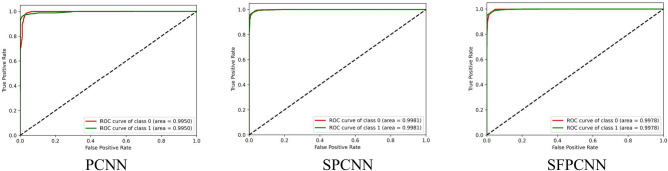
Class-wise ROCs of the three implemented methods on an external dataset of Plasmodium falciparum-infected samples.

**Fig. 29:**
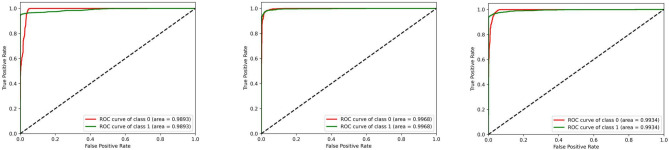
Class-wise ROCs of the three implemented methods on an external dataset of Plasmodium falciparum-infected samples.

In the dataset of Plasmodium falciparum-infected samples, SPCNN attained an average precision of around 98.23% and an accuracy of 98.80%, as indicated in Table 19, with an AUC of 99.81%. SFPCNN attained a second-place ranking with an accuracy of approximately 98.07%, while PCNN demonstrated marginally inferior performance, securing an accuracy of 97.59% and an AUC of 99.50%. The assessment of the dataset comprising Plasmodium vivax-infected samples, as outlined in Table 20, revealed SPCNN’s exceptional performance, achieving an average precision of 98.5%, an accuracy of 98.64%, and an AUC of 99.68%. Figures 28 and 29 present the class-specific ROC curves for each model across datasets comprising samples infected with Plasmodium falciparum and Plasmodium vivax, respectively, further demonstrating SPCNN’s remarkable ability to accurately differentiate between infected and healthy samples across various Plasmodium species in malaria classification.

**Table 19:** Comparison of performance metrics of the three implemented methods on an external dataset of Plasmodium falciparum-infected samples (*Bold values indicate best results).

Implemented methods	Classes	Precision (%)	Recall (%)	F1-score (%)	Accuracy (%)	AUC (%)
PCNN	Infected (0)	99.33	97.36	98.33	97.59	99.50
	Uninfected (1)	93.22	98.21	95.65		
	Average (µ) $\pm$ SD ($\sigma$) $( \%)$	96.28 $\pm 3.1$	97.79 $\pm .42$	97 $\pm .1.34$		
SPCNN	Infected (0)	99.50	98.84	99.17	**98.80**	**99.81**
	Uninfected (1)	96.96	98.67	97.81		
	Average (µ) $\pm$ SD ($\sigma$) $( \%)$	**98.23** $\pm$ **1.27**	**98.76 **$\pm$**0.08**	**98.49 **$\pm$**0.6**		
SFPCNN	Infected (0)	99.50	97.85	98.67	98.07	99.78
	Uninfected (1)	94.44	98.66	96.51		
	Average (µ) $\pm$ SD ($\sigma$) $( \%)$	96.97 $\pm 2$.53	98.25 $\pm .4$	97.57 $\pm 1.1$

**Table 20:** Comparison of performance metrics of the three implemented methods on an external dataset of Plasmodium vivax-infected samples.

Implemented methods	Classes	Precision (%)	Recall (%)	F1-score (%)	Accuracy (%)	AUC (%)
PCNN	Infected (0)	96.64	95.83	96.23	95.42	98.93
	Uninfected (1)	93.56	94.78	94.17		
	Average (µ) $\pm$ SD ($\sigma$) $( \%)$	95.10 $\pm$ 1.54	$95.31\pm$0.53	$95.2\pm$ 1.03		
SPCNN	Infected (0)	99.16	98.61	98.89	**98.64**	**99.68**
	Uninfected (1)	97.84	98.70	98.27		
	Average (µ) $\pm$ SD ($\sigma$) $( \%)$	**98.5** $\pm .67$	**98.65 **$\pm$**0.04**	**98.58 **$\pm$**0.31**		
SFPCNN	Infected (0)	98.88	98.33	98.61	98.31	99.34
	Uninfected (1)	97.41	98.26	97.84		
	Average (µ) $\pm$ SD ($\sigma$) $( \%)$	98.14 $\pm .$ 73	$98.29\pm .$ 03	98.23 $\pm .39$

### Model Interpretability

**Fig. 30:**
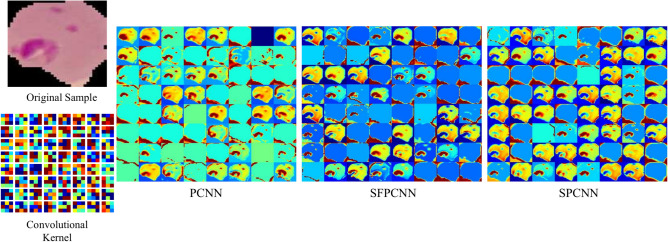
Feature maps generated by PCNN, SFPCNN, and SPCNN for malaria parasite classification.

Figure 30 shows the input of a red blood cell (RBC), likely from a blood smear used for malaria detection, alongside activation maps generated by three models, PCNN, SFPCNN, and SPCNN, as they process the image through convolutional layers. The input image in the top-left corner depicts a clear view of the RBC infected by a parasite. The grids of activations following it represent how each model’s filters respond to the image at different stages of the feature extraction process^57^. The leftmost grid, from PCNN, shows more diffuse and less focused feature maps, indicating that the model struggles to capture essential details. In the middle, SFPCNN produces more refined activations, focusing better on key areas of interest, though some features remain less sharply localized. The rightmost grid, generated by SPCNN, displays the most precise and concentrated activations, effectively highlighting the relevant regions within the cell and capturing the critical features associated with malaria infection. Based on these visualizations, SPCNN appears to extract the best features, offering a more transparent and more focused representation of the essential characteristics within the image.

Grad-CAM visualization reveals that the SPCNN model distinctly and accurately highlights the parasitized regions within the input images, offering a more precise and insightful interpretation than the other CNN architectures. Grad-CAM works by computing the gradients of a target class score concerning the feature maps from the last convolutional layer^16^. Given an input image, a forward pass is performed through the network to obtain feature map activations ${{\varvec{f}}}_{{\varvec{k}}}$, Where k represents the index of the feature maps. To assess the contribution of each feature map to the target class c, the gradient of the target class score ${{\varvec{y}}}_{{\varvec{c}}}$ to the feature map ${{\varvec{f}}}_{{\varvec{k}}}$ is calculated as,

13 $$\frac{\partial {y}_{c}}{\partial {f}_{k}}$$

These gradients are then globally averaged over the spatial dimensions to compute the important weights ${\alpha }_{k}^{c}$​ for each feature map as,

14 $$the {\alpha }_{k}^{c} =\frac{1}{Z}\sum_{i}\sum_{j}\frac{\partial {y}_{c}}{\partial {f}_{k}}$$

where Z is the total number of pixels in the feature map, and $i,j$ denote the spatial dimensions. The feature maps are then weighted by their corresponding importance weights and combined to generate a class activation map^58^. The final Grad-CAM representation, ${L}_{Grad-CAM}^{c}$ is expressed as:

15 $${L}_{Grad-CAM}^{c}=ReLU\left(\sum_{k}{\alpha }_{k}^{c}{f}_{k}\right)$$

Here, the ReLU function ensures that only positive values contribute to the final map, highlighting the most relevant regions for the target class^59^. The resulting class activation map is subsequently up-sampled to the input image size, allowing for a detailed interpretation of which regions were most influential in the model’s decision-making processes. Grad-CAM visualizations revealed that SPCNN demonstrated superior accuracy in capturing relevant features.

To further explore the interpretability of the models, SHAP was employed to quantify the contribution of each input feature to the model’s predictions. SHAP is based on cooperative game theory and uses Shapley values to attribute the importance of individual input features to the model output^32^. Given an input image, SHAP calculates the marginal contribution of each pixel by iteratively masking and redistributing the model’s prediction over coalitions of input features^60^.

SHAP assigns a value to each feature by comparing the prediction made with and without the feature^61^. For each feature, the Shapley value is computed as the average marginal contribution across all possible combinations of features. Mathematically, the Shapley value for a feature $i$ is defined as:

16 $${\phi }_{i}={\sum }_{S\subseteq N\setminus \{i\}}\frac{\left|S\right|!\left(\left|N\right|-\left|S\right|-1\right)!}{\left|N\right|!}\left(f\left(S\cup \{i\}\right)-f\left(S\right)\right)$$

where S is a subset of features excluding i, N denotes the set of all features, f(S) illustrates the model’s prediction for the subset S, $f\left(S\cup i\right)$ illustrates the model’s prediction when feature i is added to S, and ∣N∣ is the total number of features.

The model’s output for a specific input x can then be expressed as:

17 $$\widehat{y}\left(x\right)={\phi }_{0}+{\sum }_{i=1}^{M}{\phi }_{i}$$

In this context, $\widehat{y}\left(x\right)$ denotes the model prediction for input x, ${\phi }_{0}$ is the base value or average model output across the dataset, and M is the total number of features.

**Fig. 31:**
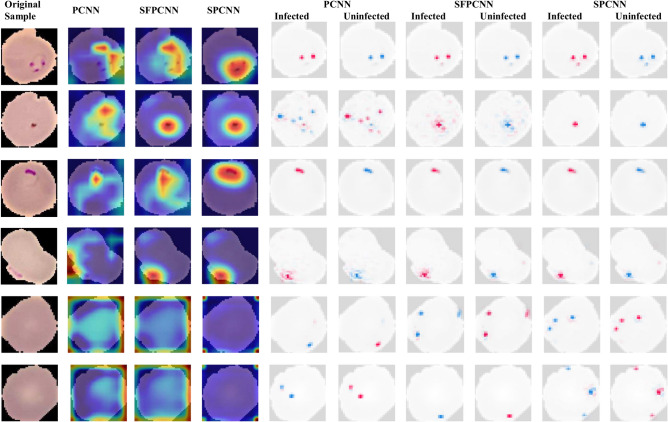
Analysis of Original RBC Images alongside Grad-CAM Visualizations and SHAP for Malaria Parasite Classification utilizing PCNN, SFPCNN, and SPCNN Models.

Using SHAP, the contributions of individual image pixels to the malaria classification task were visualized. The SHAP analysis results provide interpretive visualizations of the classification process. The SHAP images highlight regions with positive (red) or negative (blue) contributions to the model’s predictions. For example, red pixels in the top row of Fig. 31 indicate that the SPCNN model accurately classified an infected RBC sample, focusing on key parasitized regions. In contrast, the blue pixels in uninfected samples show features that reduce the likelihood of misclassification. Similarly, SHAP explanations for PCNN and SFPCNN reveal their limitations in correctly identifying parasitized areas, with more dispersed red and blue pixel clusters. For instance, PCNN misclassified the second-row image as uninfected due to its inability to focus on the parasitized region effectively. This misclassification can be attributed to the model’s tendency to spread attention across irrelevant areas, leading to weaker feature localization. The lack of precise activation around the parasitized regions suggests that PCNN struggles with complex feature patterns, particularly in cases where subtle distinctions are crucial for accurate predictions.

By combining SHAP and Grad-CAM, the analysis underscores SPCNN’s superior ability to focus on biologically relevant regions. Grad-CAM provides a high-level visual explanation of the regions of interest, while SHAP quantifies the individual pixel contributions, offering complementary perspectives on model interpretability.

## Conclusion

In this study, we investigated the efficacy of three CNN architectures—PCNN, SPCNN, and SFPCNN—for the classification of malaria parasites in RBC blood smears. Among these, the SPCNN emerged as the most effective model, demonstrating superior performance across all evaluation metrics. SPCNN achieved a precision of 99.38 $\pm$ 0.21%, recall of 99.37 $\pm$ 0.21%, F1 score of 99.37 $\pm$ 0.21%, accuracy of 99.37 ± 0.30%, and an AUC of 99.95 ± 0.01%, underscoring its robustness in accurately detecting malaria parasites. This study’s strength is its extensive evaluation of various CNN architectures designed specifically for the categorization of malaria parasites. Particularly noteworthy for its high efficiency and accuracy is the SPCNN model, which holds great promise for automated diagnosis in resource-constrained environments where accurate and timely malaria detection is essential. Nonetheless, it is important to take into account the study’s shortcomings. First off, the models may not accurately reflect the variety of blood smear samples found in various geographic locations or populations because they were trained and tested on a limited dataset. This calls into question whether other datasets can be used with the model. Furthermore, even with enhanced SPCNN performance measures, deployment on low-resource devices commonly seen in field settings may still encounter difficulties due to the network’s processing demands. In summary, SPCNN proved to be the most effective model for classifying malaria parasites, demonstrating high accuracy and robust performance metrics. The models were tested in external validation and demonstrated outstanding performance, indicating that the proposed model also excels in generalization. While the study showcases the potential of CNNs in automated diagnosis, and computational demands of the models remain areas for further exploration.

## Data Availability

Data is available on https://lhncbc.nlm.nih.gov/LHC-research/LHC-projects/image-processing/malaria-datasheet.html.

## Abbreviations

CNN: Convolutional neural network
PCNN: Parallel convolutional neural network
SPCNN: Soft attention parallel convolutional neural network
SFPCNN: Soft attention after functional block parallel convolutional neural network
Grad-CAM: Gradient-weighted class activation mapping
SHAP: Shapley additive explanations
AI: Artificial intelligence
ML: Machine learning
DL: Deep learning
WHO: World health organization
CLAHE: Contrast limited adaptive histogram equalization
ViT: Vision transformer
DeiT: Data-efficient image transformer
DB: Deep belief
ROC: Receiver operating characteristic
SGD: Stochastic gradient descent
Nadam: Nesterov-accelerated adaptive moment estimation
ADAM: Adaptive moment estimation
SOTA: State-of-the-art
